# Health outcomes of women with gestational diabetes mellitus in North West London: a 10-year longitudinal study

**DOI:** 10.1136/bmjph-2024-002279

**Published:** 2025-09-29

**Authors:** Gabrielle Goldet, Rakesh Dattani, Benjamin Pierce, Zia Ul-Haq, Tahereh Kamalati, Moulesh Shah, Andrew Frankel, Frederick Wai Keung Tam

**Affiliations:** 1 Imperial College London Department of Medicine, London, UK; 2 Imperial College Health Partners, London, London, UK; 3 Imperial College Healthcare NHS Trust, London, UK; 4 Immunology and Inflammation, Imperial College London, London, UK

**Keywords:** Cardiovascular Diseases, Reproductive History, Female, Epidemiology, Data Collection

## Abstract

**Background:**

Gestational diabetes mellitus (GDM) is known to be associated with the development of type 2 diabetes mellitus (T2DM). However, the health outcomes of women with GDM are poorly understood. The aim of this study is to better understand long-term outcomes for women having suffered GDM.

**Methods:**

Among 2.3 million people in North West London in the Discover-NOW dataset, we identified a group of 400 subjects coded with GDM between 2010 and 2011 and followed them up through to the end of 2021. Affected individuals were assessed for a variety of complications (eg, hypertension, diabetic eye disease, cerebrovascular disease, ischaemic heart disease, fatty liver disease and diabetic foot disease) and time-to-event analyses were performed.

**Results:**

The median age of first pregnancy among the study cohort was 31.57 years, with a diverse ethnic mix observed. Increased rates of T2DM, diabetic eye complications and fatty liver disease were observed. HRs (adjusted for age category, ethnicity and Index of Multiple Deprivation) were 11.32 for T2DM, 5.27 for diabetic eye complications, 7.86 for fatty liver disease and 5.69 for any comorbidity.

**Conclusion:**

We have shown the burden of multimorbidity following GDM from real-world data over a 10-year period and demonstrated high rates of diabetic eye complications and fatty liver disease that have not been shown before.

**WHAT IS ALREADY KNOWN ON THIS TOPIC**

- Gestational diabetes mellitus (GDM) is quoted as complicating the pregnancies of 4–5% of women in the UK (where screening is based on the presence of risk factors) and is becoming increasingly prevalent. The increasing GDM rate is associated with the increasing rate of obesity; the two are thought to be linked as obesity increases insulin resistance. GDM is known to confer a risk of type 2 diabetes mellitus (T2DM) (as is obesity), with an estimated third of patients with previous GDM developing T2DM within 15 years. GDM (again, like obesity) is also thought to be associated with an array of other medical conditions including the development of ischaemic heart disease and diabetic retinopathy. However, limited data currently exist exploring the long-term outcomes of individuals with GDM from the point of pregnancy onwards using large electronic data sets, which are now available.

**WHAT THIS STUDY ADDS**

- This study not only confirms known findings, such as the high rate of T2DM in women with GDM, but also highlights new data on the increased rates of diabetic eye complications and fatty liver disease compared with women whose pregnancies were not complicated by diabetes.

**HOW THIS STUDY MIGHT AFFECT RESEARCH, PRACTICE OR POLICY**

- This study highlights the significant morbidity associated with GDM and reinforces that women with GDM are a young, multimorbid group at high risk for diabetes and related complications. This emphasises the need for intensified preventative measures and adaptations to interventions like T2DM screening to better fit their everyday lives.

## Introduction

 Gestational diabetes (GDM) is quoted as complicating the pregnancies of 4–5% of women in the UK^1 2^ and is becoming increasingly prevalent.^1^ Population-based studies in the USA showed that the rate of GDM per 100 increased from 4.6 in 2006 to 8.2 in 2016.^3^ The increasing GDM rate is concurrent with an increasing rate of obesity; the two are thought to be linked, as obesity increases insulin resistance.^3 4^ In the UK, GDM is diagnosed when a patient has a fasting plasma glucose level of 5.6 mmol/L or above or a 2-hour plasma glucose level of 7.8 mmol/L or above. It is tested for early after booking in those with a previous pregnancy complicated by GDM. Otherwise, it is tested for around 20 weeks’ gestation only in those with relevant risk factors (namely, body mass index (BMI) above 30 kg/m^2^, a previous macrosomic baby weighing 4.5 kg or more, a family history of diabetes or an ethnicity with a high prevalence of diabetes). GDM is known to confer a risk of type 2 diabetes mellitus (T2DM),^5 6^ with an estimated third of patients with previous GDM developing T2DM within 15 years.^7 8^ It is associated with ischaemic heart disease,^9 10^ though there is disagreement as to whether adjustments for comorbidities such as obesity and T2DM eliminate^11 12^ or do not eliminate^13^ this effect. Recent data demonstrate an association between GDM and the long-term risk of chronic kidney disease.^14^ Over all, the sporadic data on the subject suggest a link between GDM and multimorbidity in this young cohort which warrants further exploration.

A 2017 secondary care electronic health record (EHR) study by Beharier *et al* also investigated the risk of a range of ophthalmic diseases (including diabetic retinopathy (DR) and glaucoma)^15^ after GDM. However, the rate of ophthalmic disease over a 12-year mean follow-up duration was 1.32% which appears low given the estimated risk of developing diabetes after GDM in that period would be about one third and that the yearly incidence of DR was between 8–9%^16 17^ in those with T2DM. The authors acknowledge that their secondary care data may not be capturing investigations happening outside of hospital, which could lead to a lower-than-expected rate of ophthalmic disease. In the UK, retinal screening for DR is commissioned by primary care. Primary care data are thus necessary to obtain proper capture of results of retinal screening.

Large datasets can provide invaluable information on longitudinal health outcomes in disease cohorts and, in the long term, lead to improvements in patient care.^18^ They have also been used to estimate prevalence of GDM across a whole population.^19^ However, to our knowledge, large data warehouses have not been used to examine a large panel of comorbidities of GDM over a long time period. Here, we present a retrospective longitudinal study: we report demographic data from a multiethnic cohort along with time-to-event analysis of a range of comorbidities obtained from the Discover-NOW dataset which, at the time of the study, contained the de-identified records of 2.3 million people in North West London.^18^ We thus aimed to provide a demographic picture of a group of women with GDM and explore their long-term health outcomes.

## Methods

### Study design

We used the Discover-NOW dataset to identify two groups of pregnant women: one with GDM and one without any form of diabetes, described in more detail below. The Discover-NOW dataset is a pseudoanonymised dataset (where any data which could directly be used to identify a subject have been replaced by a pseudonym) of primary care records from 365 general practitioner surgeries in North West London and is linked to secondary care data for records from 2015 onwards. During our baseline cohort entry period (1 January 2010–31 December 2011), the database contained data on around 800 000 subjects and had expanded to contain data on 2.3 million patients by the time of data collection for this study (from January 2022 to November 2022).^18^ North West London is more ethnically diverse than most of the rest of the UK and this is reflected in the database, while the age and gender distribution are broadly representative of the UK. The dataset is accessible via Discover-NOW data scientists and information governance committee-approved analysts, who are hosted by Imperial College Health Partners. We used ICD-10 and Read diagnostic codes (see online supplemental information) present in subjects’ primary and/or secondary care record to identify subjects to include in our two groups of women according to the inclusion and exclusion criteria in this study (see below). Data prior to 2015 only include primary care records as the primary and secondary care records were not linked prior to January 2015.

### Study population

**Figure 1:**
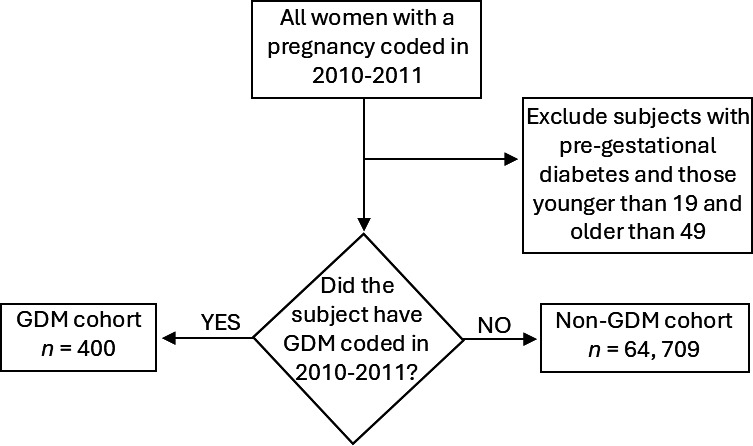
Graphical representation of cohort formation. GDM, gestational diabetes mellitus.

We identified a group of 400 women coded with (and assumed to be exposed to) GDM during a baseline entry period between 01 January 2010 and 31 December 2011 in primary care (the ‘GDM group’). We excluded men and patients with any other forms of diabetes (eg, T1DM, T2DM, drug-induced diabetes) prior to or during the baseline period. For comparison, we also identified a group of women (64 709) with a record of pregnancy in that same entry period and excluded those with a code associated with any form of diabetes—termed the ‘non-diabetic group’. A graphical representation of the formation of the two cohorts is provided in figure 1. In both groups, women aged under 19 years and over 49 years were excluded from this study as records of the timings of their pregnancies were more likely to be erroneous than those of women aged closer to the average age of pregnancy in the UK (around 30 years old); this is a similar age range to that used in other EHR studies.^8 20^ We then followed them up till 31 December 2021, death or moving to primary care practices outside of Northwest London.^21 22^

### Baseline characteristics and clinical features

Data on demographic characteristics (age during pregnancy in five year groups, smoking status (smoker, ex-smoker or non-smoker), BMI (categorised into underweight (18.5–20 kg/m^2^), normal weight (20–24 kg/m^2^), overweight (25–29 kg/m^2^) and obese (>30 kg/m^2^) according to the commonly used BMI criteria (eg, the National Institute of Clinical Evidence)) were collected during the entry period 2010–2011. The Index of Multiple Deprivation (IMD, from 1 (most deprived) to 10 (least deprived)) was derived from the 2019 release of the English Indices of Deprivation which measures levels of deprivation at the neighbourhood rather than individual level. Ethnicity data were provided by the database without a time point of the recording and were categorised using the standard categories used by the Office of National Statistics in the last census.^23^ Data on comorbidities that developed throughout the 10-year period, including hypertension, diabetic eye complications, liver disease, ischaemic heart disease, cerebrovascular disease, vascular disease and diabetic foot disease, were also collected at baseline; the clinical coding basis defining these conditions can be found in online supplemental Tables 1-3. As any subject with a diagnosis of pregestational diabetes (eg, T1DM, T2DM etc) prior to 2012 was excluded from the study, rates of diabetes codings were only collected during the follow-up period, not the baseline period. We also obtained mortality figures.

**Table 1:** Baseline characteristics of subjects presented as frequency count (percentage of total cohort)

Characteristic	Non-diabetic, N (%) n=64 709	GDM, N (%) n=400	P value
IMD decile			<0.001
1 (most deprived)	3090 (4.77)	13 (3.25)	
2	6732 (10.40)	31 (7.75)	
3	11 319 (17.49)	63 (15.75)	
4	9003 (13.91)	53 (13.24)	
5	8974 (13.87)	56 (14.00)	
6	7778 (12.02)	53 (13.25)	
7	5780 (8.93)	43 (10.75)	
8	3811 (5.89)	29 (7.25)	
9	3026 (4.68)	15 (3.75)	
10 (least deprived)	1924 (2.97)	29 (7.25)	
NULL	3272 (5.06)	14 (3.50)	
Age group (years)			<0.001*
19–24	11 295 (17.46)	28 (7.00)	
25–29	18 441 (28.50)	115 (28.75)	
30–34	19 451 (30.06)	145 (36.25)	
34–39	11 387 (17.60)	90 (22.5)	
40–44	3658 (5.65)	21 (5.25)	
45–49	477 (0.74)	<5	
NULL	0 (0.00)	0 (0.00)	
Ethnicity			<0.001
Asian or Asian British	20 010 (30.92)	211 (52.75)	
Black or black British	7944 (12.28)	63 (15.75)	
Mixed	2185 (3.38)	15 (3.75)	
Other	5437 (8.49)	28 (7.00)	
White	28 129 (43.47)	79 (19.75)	
NULL	1004 (1.55)	5 (1.25)	
BMI			<0.001
Underweight	1684 (2.6)	4 (1)	
Normal BMI	19 937 (30.8)	90 (22.5)	
Overweight	10 632 (16.4)	86 (21.5)	
Obese	6388 (9.9)	92 (23.0)	
NULL	26 068 (40.3)	128 (32.0)	
Smoking			<0.001
Ex-smoker	71.34 (11.02)	25 (6.25)	
Smoker	6758 (10.44)	20 (5.00)	
Non-smoker	1030 (1.59)	5 (1.25)	
NULL	49 787 (76.93)	350 (87.50)	

P values calculated using Chi-squared test except for the p value for age (*), which was calculated using the Fisher test

Note: as mentioned in the ‘Ethics’ section, where the frequency count was <5, it is simply stated as such.

BMI, body mass index; IMD, Index of Multiple Deprivation.

**Table 2:** Comorbidities recorded at baseline (January 2010 – December 2011)

Comorbidity	Non-diabetic, N (%) n=64, 709	GDM, N (%) n=400
Hypertension	1527 (2.36)	7 (1.75)
Eye complications	50 (0.08)	0 (0.00)
Fatty liver	91 (0.14)	<5
IHD	43 (0.07)	<5
Heart failure	31 (0.05)	<5
CVD – ischaemic	13 (0.02)	0 (0.00)
CVD – unspecified	10 (0.02)	0 (0.00)
CVD – haemorrhagic	7 (0.01)	0 (0.00)
Neuropathy	6 (0.01)	0 (0.00)
Cataract	9 (0.01)	0 (0.00)
Vascular disease	0 (0.00)	0 (0.00)
Diabetic foot disease	0 (0.00)	0 (0.00)
Amputation	0 (0.00)	0 (0.00)

CVD, cardiovascular disease; IHD, ischaemic heart disease.

### Statistical analysis

Individual characteristics are presented as frequency count (percentage), and p values demonstrating the statistical significance of the difference in the distribution of these characteristics were calculated using the χ^2^ test unless event counts were low (<5), at which point the Fisher test was used. Time-to-event analyses, in which the outcomes of interest were either a first event of any comorbidity or a first event of each individual comorbidity, included:

1. Log-rank tests: used to assess outcome differences between groups over the 10-year follow-up. Kaplan-Meier curves were generated for comorbidities where the test showed a statistically significant difference (p<0.05).
2. Cox proportional hazard regression analyses (adjusted for potentially biasing baseline characteristics: IMD decile, ethnicity and age): conducted for comorbidities with significant log-rank test differences. We adjusted for neither BMI nor smoking because of the limitations of their recording in our study (discussed in greater detail in the Results and Discussion section). Proportional hazards were confirmed by a non-significant p value from scaled Schoenfeld residuals testing.

Absence of a recording for the baseline characteristics (represented as NULL) is presented descriptively as a percentage of the total numbers in each group. As with other EHR studies,^24^ absence of a code for one of the follow-up outcome variables was assumed to indicate absence of the comorbidity rather than missing data.

Statistical testing was performed using *R* (2024-06-14, Race for Your Life) and RStudio V.2024.04.2.

## Results

### Demographics

The baseline demographics, including IMD, smoking, age, BMI and ethnicity are presented in table 1. The IMD distribution differed between the two groups, with slightly higher proportions of the non-diabetic group being in the more deprived IMD deciles than the GDM group (for example, 33% of the non-GDM group are in the lower three IMD deciles vs 27% of the GDM cohort). In terms of differences in age between the two groups, the median age was 31.57 (IQR 27.50–35.53) in the GDM group and 30.53 (IQR 26.06–33.87) in the non-diabetic group.

Documentation of ethnicity is well represented, with 98.75% of subjects in the GDM group and 98.45% in the non-diabetic group. The ethnic mixes in the two groups were quite different: the GDM group contained a higher proportion of Asian/Asian British women (52.75% vs 30.92%) and a lower proportion of white women (19.75% vs 43.47%) than the non-diabetic group.

The groups differed in BMI distributions (table 1), with a trend towards higher BMIs in the GDM group; the weighted median was 26.0 for the non-diabetic group and 28.5 for the GDM group. Exact timing of BMI measurements relative to pregnancy could not be determined, and this limitation is discussed further in the ‘Discussion’ section. There was a disparity in the proportion of subjects without recorded BMIs in the two groups, potentially biasing the results.

For smoking, no record of smoking status (either smoking, ex-smoker or non-smoking) was present in most patients (see table 1). Given the large proportion of missing data, it is not possible to draw firm conclusions on differences in smoking behaviour between the two groups.

### Comorbidities and mortality

### Baseline comorbidities

Table 2 demonstrates the comorbidities at baseline in both groups.

### Throughout follow-up: time-to-event analysis

Throughout the 10-year period, losses to follow-up occurring when subjects left their primary care practice were as follows: 95 in the GDM group and 18 826 in the non-diabetic group. These are included as censoring events in the time-to-event analysis, along with deaths: <5 subjects in the GDM group and 469 of the non-diabetic group. Mean follow-up was 4159 days in the non-diabetic cohort and 3392 days in the GDM cohort.

**Figure 2:**
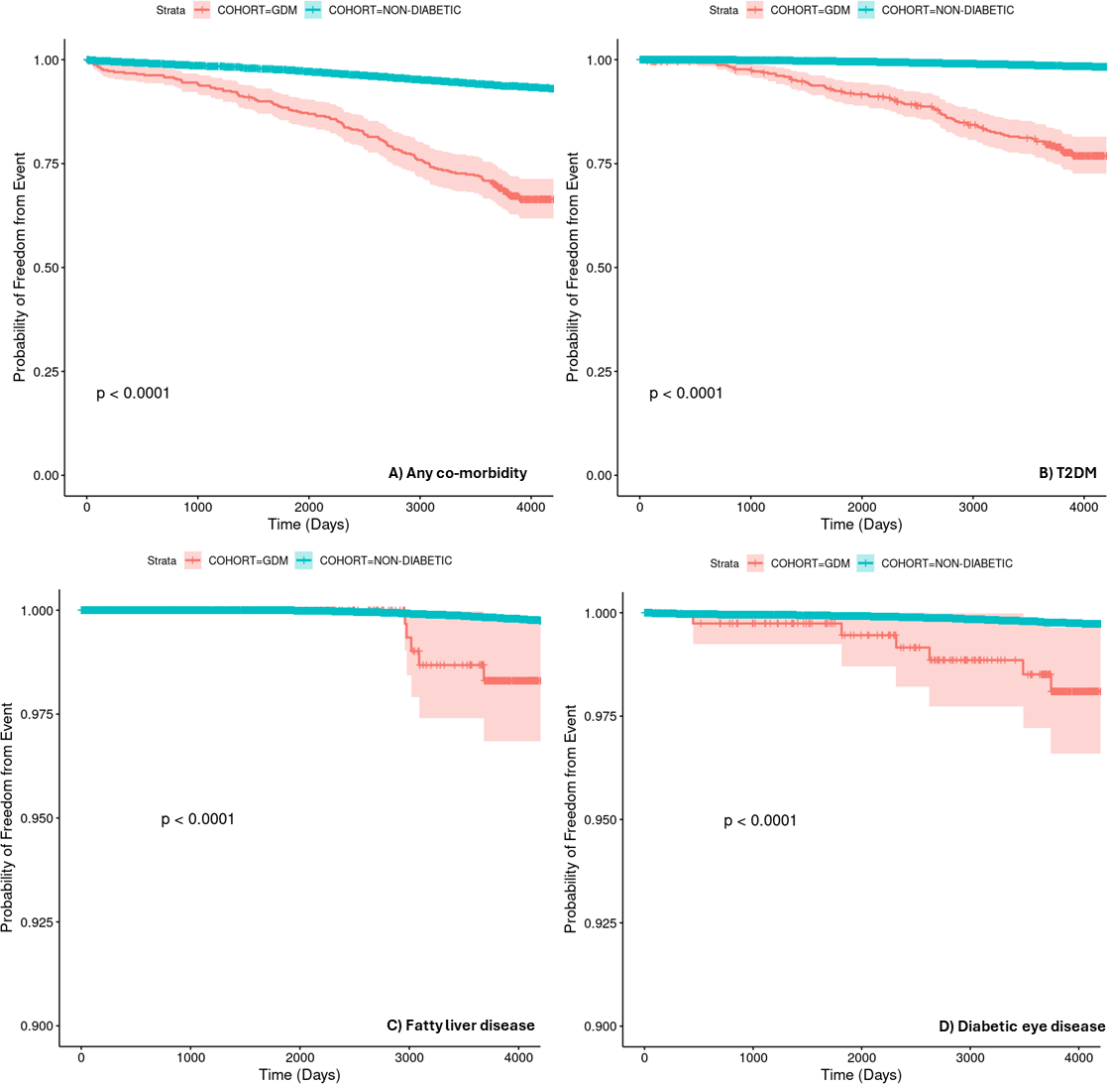
Kaplan-Meier survival curves demonstrating probability of freedom from event over time for the development of: (A) any comorbidity, (**B**) T2DM, (**C**) fatty liver disease and (D) diabetic eye disease. P value was computed using the log-rank test. Note the y-axis is expanded in C and D for clarity. GDM group (turquoise curve); non-diabetic group (orange curve). GDM, gestational diabetes mellitus; T2DM, type 2 diabetes mellitus.

We examined the event-free survival probability for developing a first event of any of the comorbidities studied. The separation in the survival curves between the two groups shown in figure 2A is distinct and provides a bold picture of overall morbidity in the GDM; this is emphasised by the HR (adjusted for IMD, age category and ethnicity) of 5.07. Table 3 shows log-rank test p values and cox proportional hazard regression results for those comorbidities for which there was a statistically significant difference in the event-free survival between the groups. BMI was not used as a covariate in Cox regression as it was deemed unreliable because of the degree of missing data and the difficulties in ascertaining when the BMI was recorded with respect to pregnancy. Similarly, smoking was not adjusted for due to a large degree of missing data.

**Table 3:** Time-to-event analysis results for the comorbidities for which there was a statistically significant event-free survival probability over the 10-year period

Comorbidity	Log-rank test P	HR (95% CI)	Cox regression P value
T2DM	<0.0001	11.10 (8.82 to 13.97)	<2×10^-16^
Diabetic eye complications	<0.0001	5.32 (2.34 to 12.09)	6×10^–5^
Fatty liver disease	<0.0001	8.50 (3.56 to 20.86)	3×10^–6^
Any comorbidity	<0.0001	5.07 (4.25 to 6.12)	<2×10^-16^

T2DM, type 2 diabetes mellitus.

Figure 2B shows the time-to-event curves for the development of T2DM in both groups, demonstrating clearly the reduction of T2DM-event-free survival in the GDM group, which is underlined by the HR of 11.10 (95% CI 8.82 to 13.97).

The assumption of proportional hazards was not met for the development of hypertension; it is therefore not appropriate to report Cox regression results for hypertension in table 3. Additionally, the p value obtained from the log-rank test for time-to-event of hypertension was non-significant (0.15). However, hypertension was the most common comorbidity in both groups at baseline and became yet more prevalent in both groups (48 out of the original 400 women in the GDM group (prior to dropouts and deaths) and 4225 out of the initial 64 709 women in the non-diabetic group developed hypertension during the 10-year follow-up period).

Diabetic eye complications overtook hypertension in the GDM group as the most common complication after T2DM. Codes related to fatty liver disease (an increasingly recognised complication of diabetes,^25^ though note disease terminology has changed recently, and this disease entity is more correctly described by the term metabolic dysfunction-associated steatotic (MASD) liver disease) were also prominent in the GDM group.

HRs for diabetic eye complications (5.32) and fatty liver (8.50) were particularly high. Event counts for all the other comorbidities studied (see online supplemental file 1) were low (particularly in the much smaller GDM cohort) and the differences in event-free survival between the groups did not reach statistical significance; these data are therefore not displayed here.

## Discussion

This work demonstrates that GDM is a risk factor for a variety of comorbidities with respect to women whose pregnancies were not complicated by GDM (see figure 2 and table 3), particularly diabetic eye disease (HR 5.32) and fatty liver disease (HR 8.50). The survival curves for fatty liver disease in figure 2C clearly separate from 3000 days onwards; this may be due to the impact of the linkage of the primary care database with the secondary care database in 2015. Though our data on T2DM may be confounded by screening for diabetes in the GDM group with a 13-week postpartum and then yearly HbA1c, the significantly higher proportion of subjects coded for T2DM in the GDM group versus the non-diabetic group in the longitudinal follow-up agrees with prior studies highlighting the greater risk of T2DM in women with a history of GDM.^7^ This study’s HR (11.10) for T2DM agrees with published research (eg, a range of 3.87^26^ to 14.2 from systematic review data^7^); this attests to the robustness of our results. Our study thus highlights the need for interventions to promote screening for diabetes, such as sending proactive invitations for individuals with a prior history of GDM for screening and offering flexible screening times, as suggested by Dennison *et al*.^27^

Our high prevalence of fatty liver disease (HR=8.50) adds to evidence from case-controlled studies on the risk of long-term fatty liver disease^28^ and presents new results in terms of the high rate of diabetic eye complications (HR=5.32) in the longitudinal follow-up among the GDM group.

### Strengths and limitations

The strength of this retrospective longitudinal study is how the use of data science has allowed us to elucidate the demographic characteristics of women with GDM and determine their risk of multimorbidity over a 10-year period. Our study is distinct as it excludes women with pregestational diabetes from their comparison groups and follows them from the point of pregnancy onwards; though two UK Biobank studies examine long-term cardiovascular disease^29^ and mortality,^30^ they examine long-term outcomes years after self-reported pregnancies from questionnaires rather than longitudinal follow-up from the point of pregnancy from actual primary care and secondary care records. In Lee *et al*’s study,^29^ women were enrolled on average 25–30 years after their first live birth and in Michalopolou *et al*, women were enrolled at least 12 years after their first live birth.^30^ The study uniquely highlights the difference in outcomes between women with GDM and those whose pregnancies were not complicated by diabetes from their pregnancy onwards. To our knowledge, this has not been done before.

This work is a preliminary retrospective cohort study (given the difference in size of the groups) and acts as a platform for guiding future investigations about particular complications of note in women with a background of GDM and is to be followed up with formal cohort studies.

Any study using Big Data as a measure of population statistics is limited by the accuracy and completeness of coding and of diagnosis. This is particularly notable in the absence of coding for baseline characteristics (eg, smoking status); indeed, previous EHR research in the UK has highlighted imperfect coding practice on smoking status.^31^ There was a higher proportion of subjects lacking a recording of baseline comorbidities (apart from smoking) in the non-diabetic group (see table 1). This may result from this being a group of relatively healthy women whose records are likely to be less complete than women in the GDM group who will have much more contact with health services. The potentially higher degree of missingness in codings of baseline and outcome data in the non-diabetic group may bias the analysis, favouring ostensibly higher rates of both baseline and follow-up co-morbidities in the GDM group.

Codes pertaining to termination of pregnancy and pregnancy loss were included in our pregnancy inclusion code list (online supplemental table 1) to improve our capture of pregnancy events. It should be noted, however, that unless a woman has had GDM in a previous pregnancy, it is only tested for at 20 weeks of gestation, but most spontaneous or induced abortions occur in the first trimester. This means that some subjects whose pregnancies ended early are included in the non-diabetic group, but had they progressed to the point of testing for GDM, they may have potentially been found to have the condition. However, if GDM had been present and not yet diagnosed in some subjects included in the non-diabetic cohort, the impact, in terms of long-term complications, would be expected to be lower given that the hyperglycaemic state was experienced for a shorter period, and therefore this detection bias should not have a significant effect.

Screening for GDM is also likely to confound our relative ethnic mixes in the two groups. This is because, in the UK, only women with certain risk factors (including Asian ethnicity) are screened for GDM, as it is well-known that there are ethnic differences between women with GDM and pregnant women without GDM.^32^ Thus, an Asian woman is more likely to be screened for and diagnosed with GDM. In addition, there is likely to be bias in the difference in prevalence of T2DM between the GDM and non-diabetic groups on account of the yearly screening for T2DM that women with previous GDM are supposed to have. On the other hand, it is well-established that the diabetes screening programme after GDM has variable take-up,^8^ so we may in fact be underestimating the true prevalence of T2DM in the GDM group.

Screening for DR only occurs once one is diagnosed with diabetes. The higher rate of diagnosis of diabetes among women with GDM is biased by screening for diabetes; this higher rate of diabetes diagnoses in the GDM group will have a consequent amplifying effect on the rate of diabetic eye problems in this group relative to the non-diabetic group. Surveillance bias, given the yearly check-ups women with diabetes receive, is also likely to affect other outcomes such as cardiovascular disease and MASD. For example, women with diabetes have yearly blood pressure and lipid profiles checked, and their increased contact with primary and secondary care services augments the likelihood of MASD being detected.

GDM is defined as any form of diabetes arising in pregnancy, whether it be in fact previously undiagnosed pregestational diabetes or true GDM that resolves postpartum. Therefore, another confounder is the likely small number of cases recorded as GDM in pregnant women who in fact have another form of diabetes that is incidentally picked up in pregnancy.

As with other UK database studies comparing women with a past of GDM versus pregnant women without diabetes, the proportion of women in our GDM group was low (0.62%, c.f. 0.64% in one UK Biobank study^29^ and 0.55% in another).^30^ Undercoding of GDM is known to be a problem in the UK, and the Maternity Services Dataset used by the National Gestational Diabetes Audit is described as ‘immature’ with ‘suboptimal’ data quality on GDM.^33^ One factor which may be related to the small size of the GDM group and cause bias in the relative outcomes of the two groups is whether our GDM cohort contains only a select group of women with GDM (for example, those with other comorbidities, as opposed to those with fewer or no other medical problems are more likely to interact with GP services more, have more complete records and therefore an update of their GDM status rather than just a record of their pregnancy). This would be expected to bias the results by increasing the HRs for complications in the GDM group. Additionally, the primary and secondary care databases were linked in 2015. During our baseline entry period, the database had therefore not yet been linked to the secondary care database. All results prior to 2015 reported here are from primary care only, whereas those thereafter emanate from primary *and* secondary care. GDM is a condition largely treated in hospitals (secondary care). The lack of access to secondary care data during the baseline period may have the following effects:

- Women with GDM followed up in hospital may not have had their pregnancy records transferred to primary care, excluding them from both groups. This reduces the GDM cohort size and increases the CIs for this group.
- Women with GDM followed up only in hospitals may have had their pregnancy recorded in primary care but not their GDM status, placing them in the non-diabetic group. This could lessen the difference between groups and, as above, reduce the GDM cohort size and widen CIs.

Here, we only used the diagnostic code ‘gestational diabetes’ to define our GDM group, which may also contribute to the lower-than-expected proportion of women in the GDM group relative to the non-diabetic group. For example, had we corroborated a date of delivery with an oral glucose tolerance test diagnostic of GDM, we might have increased the numbers of subjects in our GDM group. This, however, relies on the timeliness of coding of the oral glucose tolerance test and the date of delivery. Thus, the advantage of only selecting subjects coded with GDM is that it is more likely to represent an actual diagnosis of GDM. However, to improve sensitivity in future studies, a broader code list for the definition of GDM will be used for comparison.

Our data on BMI should be interpreted with caution as measurements of BMI around the time of pregnancy may be affected by gestational weight gain. This issue should, however, affect both groups and would only be a confounder if gestational weight gain was different in the two groups, that is, if it was affected by GDM. BMI is also an important confounder in our cardiovascular outcomes, with higher BMI being a known risk factor for cardiovascular disease and GDM.^34^

Timeliness of coding is also an important factor. For example, women in much older age groups than are included here were found to have codes associated with pregnancy during the baseline entry period in our initial data extraction. This is clearly not reflective of them being pregnant during our study period, but rather this is suggestive of them having been coded for *previous* pregnancies during our study period at which point they were of more advanced age and the pregnancy was long behind them. We have thus contained the study to age groups in which pregnancy is more common. Indeed, the median ages of pregnancy in our study in the GDM group (31.57) and the non-diabetic group (30.53) are similar to the 2011 UK mean age of delivery (29.5), thus demonstrating the validity of this approach.

50 subjects in the non-diabetic group (0.08%) were coded with diabetic eye disease, a coding that may plausibly be diagnostic of diabetes itself, in the baseline entry period. This is, however, a very small proportion of the cohort which is not expected to greatly affect results. Future studies will corroborate such codings with other signs of diabetes, such as records of medication or tests for glycaemia.

Finally, the nature and size of the effect of the COVID-19 pandemic on clinical coding (not to mention diagnosis) is yet to be fully understood and will have an impact on the last 2 years of the follow-up.

### Future work

Other covariates than those used here will impact on the risk of our outcomes in regression analysis. Notably, T2DM, BMI and hypertension are risk factors for cardiovascular and vascular disease. With a larger cohort size, distinguishing the subtle effects of these time-varying covariates will provide an even more accurate picture of the association between GDM and its possible complications. This study highlights the need for a larger cohort study (with larger event rates) which will help to decipher the effects of comorbidities on other comorbidities occurring in the longitudinal follow-up.

Further investigations are ongoing on the high rate of diabetic eye complications in our population. If the rate of DR is demonstrated to be comparable to that observed in women with other forms of diabetes (be they pregnant or not) who *are* eligible for eye screening, then this suggests women with GDM should be eligible for eye screening postpartum as well as their 13-week and yearly HbA1cs. This is because studies have shown that up to 21% of patients with T2DM had DR by the time of diagnosis of diabetes^35^ and that at first screening within the year since diagnosis of T2DM, 19% of patients have some degree of DR.^36^ This suggests that by the time people are diagnosed with diabetes, some are likely to already have retinopathic changes. Of course, this may simply be because of late diagnosis of diabetes during the study period, which would be obviated by effective screening for diabetes among women with previous GDM with high uptake. However, it may be that there is evidence of retinal damage from when diabetes starts to occur prior to formal serological diagnosis of diabetes. Indeed, non-diabetic hyperglycaemia, which precedes diabetes, is known to be associated with DR^37^; GDM could impact on this risk in a similar way. A diagnosis of GDM could thus be seen as a flag pointing to individuals at risk of incipient DR in whom early retinal screening should be considered and, in cases where DR has been detected prior to a serological diagnosis of diabetes, indicate that more intensive monitoring of glycaemia is necessary. There is increasing evidence that artificial intelligence can be leveraged to aid diagnosis of DR,^38^ and with attachments to smartphones that can be used for funduscopy, a future in which women are able to undertake their own retinal screening in the convenience of their homes is plausible, and would improve ease of screening of a group known to find diabetes screening difficult to attend.^27^ This would, in turn, improve surveillance of other cardiovascular complications in this group of women.

Additionally, other conditions which are slower to progress may require a longer follow-up than we have undertaken here to demonstrate a signal. For example, there are reports of GDM and glucose intolerance in pregnancy potentially being a risk factor for malignancy.^39^,^42^ A longer follow-up period may be more appropriate to detect the development of this condition.

## Conclusion

This study presents new data on the increased rates of diabetic eye complications and fatty liver disease compared with non-diabetic pregnancies and supports evidence of the high rate of T2DM in women with GDM. While previous studies focused on ophthalmological risks in secondary care, our inclusion of primary care data provides a more comprehensive view and is thus more generalisable. The findings reinforce that women with GDM are a young, multimorbid group at high risk for diabetes and related complications, emphasising the need for intensified preventative measures and adaptations to interventions like T2DM screening to better fit their everyday lives.

## Supplementary material

10.1136/bmjph-2024-002279 online supplemental file 1

## Data Availability

All data relevant to the study are included in the article or uploaded as supplementary information.
