# Supplementary material for: Health outcomes of women with gestational diabetes mellitus in North West London: a 10-year longitudinal study
Source: BMJ Public Health. 2025 Sep 29;3(2):e002279. doi: 10.1136/bmjph-2024-002279 (PMC12481313; doi:10.1136/bmjph-2024-002279)
Supplement: online supplemental file 1 [file bmjph-3-2-s001.docx]

Supplemental Information

# Diagnostic code list

## Table 1: Cohort inclusion codes

| **Diagnostic code** | **Description** | **Cohort** | **Code type** |
| --- | --- | --- | --- |
| 1514 | Estimated date of delivery | Non-diabetic | CTV3 |
| 153.. | Gravida | Non-diabetic | CTV3 |
| 27Z.. | Obstetric examination NOS | Non-diabetic | CTV3 |
| 62… | Patient pregnant | Non-diabetic | CTV3 |
| 621.. | Patient currently pregnant | Non-diabetic | CTV3 |
| 62B1. | Delivery: no place booked | Non-diabetic | CTV3 |
| 62N.. | Antenatal examination | Non-diabetic | CTV3 |
| 7.00E+91 | Termination of pregnancy 7E091 | Non-diabetic | CTV3 |
| 7F12. | Elective caesarean section | Non-diabetic | CTV3 |
| 7F133 | Emergency caesarean section | Non-diabetic | CTV3 |
| 7F14. | Breech extraction delivery | Non-diabetic | CTV3 |
| 7F150 | Spontaneous breech delivery | Non-diabetic | CTV3 |
| 7F17. | Vacuum delivery | Non-diabetic | CTV3 |
| 7F19. | Normal delivery | Non-diabetic | CTV3 |
| 8HT9. | Referral to antenatal clinic | Non-diabetic | CTV3 |
| L02.. | Missed abortion | Non-diabetic | CTV3 |
| L03.. | Ectopic pregnancy | Non-diabetic | CTV3 |
| L04.. | Spontaneous abortion L04.. | Non-diabetic | CTV3 |
| L100. | Threatened miscarriage | Non-diabetic | CTV3 |
| L1000 | Threatened abortion unspecified | Non-diabetic | CTV3 |
| L1002 | Threatened abortion –not delivered | Non-diabetic | CTV3 |
| L100z | Threatened abortion NOS | Non-diabetic | CTV3 |
| L12B. | Proteinuric hypertension of pregnancy | Non-diabetic | CTV3 |
| L162. | Albuminuria in pregnancy without hypertension | Non-diabetic | CTV3 |
| L1632 | Habitual aborter - not delivered | Non-diabetic | CTV3 |
| L1633 | Pregnancy care of habitual aborter | Non-diabetic | CTV3 |
| L16C. | Pregnancy induced oedema+proteinuria without hypertension | Non-diabetic | CTV3 |
| L20.. | Normal delivery in a completely normal case | Non-diabetic | CTV3 |
| L21.. | Multiple gestation | Non-diabetic | CTV3 |
| L210. | Twin pregnancy | Non-diabetic | CTV3 |
| L211. | Triplet pregnancy | Non-diabetic | CTV3 |
| L212. | Quadruplet pregnancy | Non-diabetic | CTV3 |
| L21y. | Other multiple pregnancy | Non-diabetic | CTV3 |
| L21z. | Multiple pregnancy NOS | Non-diabetic | CTV3 |
| L264. | Intrauterine death | Non-diabetic | CTV3 |
| L395. | Forceps delivery | Non-diabetic | CTV3 |
| L396. | Vacuum extractor delivery | Non-diabetic | CTV3 |
| L3983 | Delivery by elective caesarean section | Non-diabetic | CTV3 |
| L3984 | Delivery by emergency caesarean section L3984 | Non-diabetic | CTV3 |
| Ly0.. | Spontaneous vertex delivery | Non-diabetic | CTV3 |
| Ly1.. | Spontaneous breech delivery Ly1, 7F150 | Non-diabetic | CTV3 |
| Lyu1. | [X]Oedema,proteinuria+hypertens in pregnancy,childbrth,puerp | Non-diabetic | CTV3 |
| Lyu30 | [X]Other multiple gestation | Non-diabetic | CTV3 |
| X40Ak | Undiagnosed multiple pregnancy | Non-diabetic | CTV3 |
| X40Ar | Viable pregnancy | Non-diabetic | CTV3 |
| X40Az | Quintuplet pregnancy | Non-diabetic | CTV3 |
| X40B0 | Sextuplet pregnancy | Non-diabetic | CTV3 |
| X40B1 | Septulet pregnancy | Non-diabetic | CTV3 |
| X40B2 | Continuing pregnancy after abortion of sibling fetus | Non-diabetic | CTV3 |
| X40B3 | Continuing pregnancy after intrauterine death of sibling fetus | Non-diabetic | CTV3 |
| X40BA | Viable fetus in abdominal pregnancy | Non-diabetic | CTV3 |
| X74V6 | Antenatal care | Non-diabetic | CTV3 |
| X76Qk | Estimated date of conception | Non-diabetic | CTV3 |
| X76Qt | Confirmation of pregnancy | Non-diabetic | CTV3 |
| X76Qv | Length of gestation | Non-diabetic | CTV3 |
| Xa4SO | Observation of pattern of pregnancy | Non-diabetic | CTV3 |
| Xa4SU | Normal pregnancy | Non-diabetic | CTV3 |
| Xa85U | Intrauterine pregnancy | Non-diabetic | CTV3 |
| XaKXd | Refer to early pregnancy unit | Non-diabetic | CTV3 |
| XaXfi | Referral to teenage pregnancy and parenting support service | Non-diabetic | CTV3 |
| XaXUk | Self referral to termination of pregnancy service | Non-diabetic | CTV3 |
| XM1Da | Examination of uterine fundus size | Non-diabetic | CTV3 |
| XSLD5 | Gestational age | Non-diabetic | CTV3 |
| 4453 | Serum pregnancy test positive | Non-diabetic | CTV3 |
| 4654 | Urine pregnancy test positive | Non-diabetic | CTV3 |
| 584D. | A/N u/s confirm i-u pregnancy | Non-diabetic | CTV3 |
| 6222 | Antenatal care: 2nd pregnancy | Non-diabetic | CTV3 |
| 6223 | Antenatal care: 3rd pregnancy | Non-diabetic | CTV3 |
| 624.. | A/N care: precious pregnancy | Non-diabetic | CTV3 |
| 62a.. | Pregnancy review | Non-diabetic | CTV3 |
| 62O7. | Pregnancy prolonged - 41 weeks | Non-diabetic | CTV3 |
| 62O8. | Pregnancy prolonged - 42 weeks | Non-diabetic | CTV3 |
| 676.. | Pre-pregnancy counselling | Non-diabetic | CTV3 |
| 6761 | Diabetic pre-pregnancy counsel | Non-diabetic | CTV3 |
| 67A.. | Pregnancy advice | Non-diabetic | CTV3 |
| 67A2. | Diet in pregnancy advice | Non-diabetic | CTV3 |
| 67A3. | Pregnancy smoking advice | Non-diabetic | CTV3 |
| 67A4. | Pregnancy exercise advice | Non-diabetic | CTV3 |
| 67A5. | Pregnancy alcohol advice | Non-diabetic | CTV3 |
| 67A6. | Drugs in pregnancy advice | Non-diabetic | CTV3 |
| 67A7. | Pregnancy dental advice | Non-diabetic | CTV3 |
| 12G1. | FH: Raised B.P. in pregnancy | Non-diabetic | CTV3 |
| 12G2. | FH: Diabetes in pregnancy | Non-diabetic | CTV3 |
| 12G4. | FH: Multiple pregnancy | Non-diabetic | CTV3 |
| 7.00E+66 | Hysterot termination pregnancy | Non-diabetic | CTV3 |
| 7.00E+84 | Suction termination pregnancy | Non-diabetic | CTV3 |
| 8B74. | Iron supplement in pregnancy | Non-diabetic | CTV3 |
| 8B75. | Vitamin supplement - pregnancy | Non-diabetic | CTV3 |
| 8Cg.. | Pregnancy termination care | Non-diabetic | CTV3 |
| 8HHf. | Refer to early pregnancy unit | Non-diabetic | CTV3 |
| 8HHV. | Ref for termination pregnancy | Non-diabetic | CTV3 |
| 8M6.. | Requests pregnancy termination | Non-diabetic | CTV3 |
| 9NkN. | Seen in early pregnancy unit | Non-diabetic | CTV3 |
| L.... | Pregnancy/childbrth/puerperium | Non-diabetic | CTV3 |
| L0... | Pregnancy + abortive outcome | Non-diabetic | CTV3 |
| L03.. | Ectopic pregnancy | Non-diabetic | CTV3 |
| L030. | Abdominal pregnancy | Non-diabetic | CTV3 |
| L031. | Tubal pregnancy | Non-diabetic | CTV3 |
| L0310 | Fallopian tube pregnancy | Non-diabetic | CTV3 |
| L031z | Tubal pregnancy NOS | Non-diabetic | CTV3 |
| L032. | Ovarian pregnancy | Non-diabetic | CTV3 |
| L03y. | Other ectopic pregnancy | Non-diabetic | CTV3 |
| L03y0 | Cervical pregnancy | Non-diabetic | CTV3 |
| L03y1 | Cornual pregnancy | Non-diabetic | CTV3 |
| L03y2 | Membranous pregnancy | Non-diabetic | CTV3 |
| L03y3 | Combined/heterotopic pregnancy | Non-diabetic | CTV3 |
| L03y4 | Mural pregnancy | Non-diabetic | CTV3 |
| L03y5 | Intraligamentous pregnancy | Non-diabetic | CTV3 |
| L03y6 | Mesenteric pregnancy | Non-diabetic | CTV3 |
| L03y7 | Angular pregnancy | Non-diabetic | CTV3 |
| L03y8 | Mesometric pregnancy | Non-diabetic | CTV3 |
| L03yz | Other ectopic pregnancy NOS | Non-diabetic | CTV3 |
| L03z. | Ectopic pregnancy NOS | Non-diabetic | CTV3 |
| L0y.. | Pregnancy+abortive outcome OS | Non-diabetic | CTV3 |
| L0z.. | Pregnancy+abortive outcome NOS | Non-diabetic | CTV3 |
| L1... | Pregnancy complications | Non-diabetic | CTV3 |
| L10.. | Haemorrhage in early pregnancy | Non-diabetic | CTV3 |
| L10y. | Other early pregnancy haem'ge | Non-diabetic | CTV3 |
| L10yz | Other early pregnancy h'ge NOS | Non-diabetic | CTV3 |
| L10z. | Early pregnancy haemorrh. NOS | Non-diabetic | CTV3 |
| L10z0 | Early pregnancy h'ge NOS unsp. | Non-diabetic | CTV3 |
| L10zz | Early pregnancy h'ge NOS | Non-diabetic | CTV3 |
| L1265 | Eclampsia in pregnancy | Non-diabetic | CTV3 |
| L12B. | Protein hypertens of pregnancy | Non-diabetic | CTV3 |
| L13.. | Excessive pregnancy vomiting | Non-diabetic | CTV3 |
| L132. | Late vomiting of pregnancy | Non-diabetic | CTV3 |
| L132z | Late pregnancy vomiting NOS | Non-diabetic | CTV3 |
| L13y. | Other pregnancy vomiting | Non-diabetic | CTV3 |
| L13yz | Other pregnancy vomiting NOS | Non-diabetic | CTV3 |
| L13z. | Unspecified pregnancy vomiting | Non-diabetic | CTV3 |
| L13zz | Unspec.pregnancy vomiting NOS | Non-diabetic | CTV3 |
| L15.. | Prolonged/post-term pregnancy | Non-diabetic | CTV3 |
| L150. | Post-term pregnancy | Non-diabetic | CTV3 |
| L1500 | Post-term pregnancy-unspecif. | Non-diabetic | CTV3 |
| L1501 | Post-term pregnancy-delivered | Non-diabetic | CTV3 |
| L1502 | Post-term pregnancy-not deliv. | Non-diabetic | CTV3 |
| L150z | Post-term pregnancy NOS | Non-diabetic | CTV3 |
| L15z. | Prolonged pregnancy NOS | Non-diabetic | CTV3 |
| L16.. | Other pregnancy complic. NEC | Non-diabetic | CTV3 |
| L164. | Peripheral neuritis+pregnancy | Non-diabetic | CTV3 |
| L164z | Periph.neuritis+pregnancy NOS | Non-diabetic | CTV3 |
| L1667 | Infctn/genital tract/pregnancy | Non-diabetic | CTV3 |
| L1668 | UTI complicating pregnancy | Non-diabetic | CTV3 |
| L167. | Pregnancy + liver disorder | Non-diabetic | CTV3 |
| L167z | Pregnancy + liver disorder NOS | Non-diabetic | CTV3 |
| L168. | Fatigue during pregnancy | Non-diabetic | CTV3 |
| L1680 | Fatigue + pregnancy unspecif. | Non-diabetic | CTV3 |
| L1681 | Fatigue + pregnancy-delivered | Non-diabetic | CTV3 |
| L1683 | Fatigue+pregnancy - not deliv. | Non-diabetic | CTV3 |
| L1684 | Fatigue+pregnancy+p/n complic. | Non-diabetic | CTV3 |
| L168z | Fatigue during pregnancy NOS | Non-diabetic | CTV3 |
| L16A. | Glycosuria during pregnancy | Non-diabetic | CTV3 |
| L16A0 | Glycosuria in pregnancy-unsp. | Non-diabetic | CTV3 |
| L16Az | Glycosuria in pregnancy NOS | Non-diabetic | CTV3 |
| L16E. | Pregnancy pruritus | Non-diabetic | CTV3 |
| L16y. | Other pregnancy complications | Non-diabetic | CTV3 |
| L16y5 | Abdominal pain in pregnancy | Non-diabetic | CTV3 |
| L16yz | Other pregnancy complicat.NOS | Non-diabetic | CTV3 |
| L16z. | Pregnancy complication NOS | Non-diabetic | CTV3 |
| L170. | Pregnancy + syphilis | Non-diabetic | CTV3 |
| L1700 | Pregnancy+syphilis unspecified | Non-diabetic | CTV3 |
| L1701 | Pregnancy+syphilis-delivered | Non-diabetic | CTV3 |
| L170z | Pregnancy + syphilis NOS | Non-diabetic | CTV3 |
| L171. | Pregnancy + gonorrhoea | Non-diabetic | CTV3 |
| L171z | Pregnancy + gonorrhoea NOS | Non-diabetic | CTV3 |
| L173. | Pregnancy + tuberculosis | Non-diabetic | CTV3 |
| L173z | Pregnancy + tuberculosis NOS | Non-diabetic | CTV3 |
| L174. | Pregnancy + malaria | Non-diabetic | CTV3 |
| L1740 | Pregnancy+malaria unspecified | Non-diabetic | CTV3 |
| L1741 | Pregnancy+malaria-delivered | Non-diabetic | CTV3 |
| L1742 | Pregnancy+malaria-del+p/n comp | Non-diabetic | CTV3 |
| L1743 | Pregnancy+malaria-not deliv. | Non-diabetic | CTV3 |
| L1744 | Pregnancy+malaria+p/n complic. | Non-diabetic | CTV3 |
| L174z | Pregnancy + malaria NOS | Non-diabetic | CTV3 |
| L175. | Pregnancy + rubella | Non-diabetic | CTV3 |
| L1750 | Pregnancy+rubella unspecified | Non-diabetic | CTV3 |
| L1751 | Pregnancy+rubella-delivered | Non-diabetic | CTV3 |
| L1752 | Pregnancy+rubella-del+p/n comp | Non-diabetic | CTV3 |
| L1753 | Pregnancy+rubella-not deliv. | Non-diabetic | CTV3 |
| L1754 | Pregnancy+rubella+p/n complic. | Non-diabetic | CTV3 |
| L175z | Pregnancy + rubella NOS | Non-diabetic | CTV3 |
| L176. | Pregnancy+other viral diseases | Non-diabetic | CTV3 |
| L176z | Pregnancy+other viral dis.NOS | Non-diabetic | CTV3 |
| L177. | Infect of bladder in pregnancy | Non-diabetic | CTV3 |
| L178. | Infection urethra in pregnancy | Non-diabetic | CTV3 |
| L180. | Pregnancy + diabetes mellitus | Non-diabetic | CTV3 |
| L1808 | Diabetes mel aris in pregnancy | Non-diabetic | CTV3 |
| L181. | Pregnancy+thyroid dysfunction | Non-diabetic | CTV3 |
| L182. | Pregnancy + anaemia | Non-diabetic | CTV3 |
| L1820 | Pregnancy+anaemia unspecified | Non-diabetic | CTV3 |
| L1821 | Pregnancy+anaemia-delivered | Non-diabetic | CTV3 |
| L1822 | Pregnancy+anaemia-del+p/n comp | Non-diabetic | CTV3 |
| L1823 | Pregnancy+anaemia-not deliver. | Non-diabetic | CTV3 |
| L1824 | Pregnancy+anaemia+p/n complic. | Non-diabetic | CTV3 |
| L1825 | Iron def anaemia of pregnancy | Non-diabetic | CTV3 |
| L182z | Pregnancy + anaemia NOS | Non-diabetic | CTV3 |
| L183. | Pregnancy+drug dependence | Non-diabetic | CTV3 |
| L184. | Pregnancy + mental disorders | Non-diabetic | CTV3 |
| L184z | Pregnancy+mental disorders NOS | Non-diabetic | CTV3 |
| L185. | Pregnancy + congenital CVS dis | Non-diabetic | CTV3 |
| L186. | Pregnancy+other CVS diseases | Non-diabetic | CTV3 |
| L186z | Pregnancy+other CVS dis. NOS | Non-diabetic | CTV3 |
| L188z | Pregnancy + abnormal GTT NOS | Non-diabetic | CTV3 |
| L18A0 | Cholestasis of pregnancy | Non-diabetic | CTV3 |
| L1y.. | Pregnancy complications OS | Non-diabetic | CTV3 |
| L1z.. | Pregnancy complications NOS | Non-diabetic | CTV3 |
| L2... | Risk factors in pregnancy | Non-diabetic | CTV3 |
| L21.. | Multiple pregnancy | Non-diabetic | CTV3 |
| L210. | Twin pregnancy | Non-diabetic | CTV3 |
| L2100 | Twin pregnancy unspecified | Non-diabetic | CTV3 |
| L2101 | Twin pregnancy - delivered | Non-diabetic | CTV3 |
| L2102 | Twin pregnancy +antenatal prob | Non-diabetic | CTV3 |
| L210z | Twin pregnancy NOS | Non-diabetic | CTV3 |
| L211. | Triplet pregnancy | Non-diabetic | CTV3 |
| L2110 | Triplet pregnancy unspecified | Non-diabetic | CTV3 |
| L2111 | Triplet pregnancy - delivered | Non-diabetic | CTV3 |
| L211z | Triplet pregnancy NOS | Non-diabetic | CTV3 |
| L212. | Quadruplet pregnancy | Non-diabetic | CTV3 |
| L2120 | Quadruplet pregnancy unspecif. | Non-diabetic | CTV3 |
| L2121 | Quadruplet pregnancy-delivered | Non-diabetic | CTV3 |
| L212z | Quadruplet pregnancy NOS | Non-diabetic | CTV3 |
| L21y. | Other multiple pregnancy | Non-diabetic | CTV3 |
| L21y0 | Other multiple pregnancy unsp. | Non-diabetic | CTV3 |
| L21yz | Other multiple pregnancy NOS | Non-diabetic | CTV3 |
| L21z. | Multiple pregnancy NOS | Non-diabetic | CTV3 |
| L21z0 | Multiple pregnancy NOS unspec. | Non-diabetic | CTV3 |
| L21z1 | Multiple pregnancy NOS-deliv. | Non-diabetic | CTV3 |
| L21zz | Multiple pregnancy NOS | Non-diabetic | CTV3 |
| L2B.. | Low weight gain in pregnancy | Non-diabetic | CTV3 |
| L2C.. | Malnutrition in pregnancy | Non-diabetic | CTV3 |
| L2D.. | Retained IUCD in pregnancy | Non-diabetic | CTV3 |
| L2y.. | Risk factors in pregnancy OS | Non-diabetic | CTV3 |
| L2z.. | Risk factors in pregnancy NOS | Non-diabetic | CTV3 |
| L4105 | VVs of legs in pregnancy | Non-diabetic | CTV3 |
| L4115 | Genital varices in pregnancy | Non-diabetic | CTV3 |
| L4155 | Other phlebitis in pregnancy | Non-diabetic | CTV3 |
| L4166 | Haemorrhoids in pregnancy | Non-diabetic | CTV3 |
| Lyu00 | [X]Other ectopic pregnancy | Non-diabetic | CTV3 |
| M2405 | Alopecia of pregnancy | Non-diabetic | CTV3 |
| Q014. | Fetus+ectopic pregnancy | Non-diabetic | CTV3 |
| Q0141 | Fetus+abdom.ectopic pregnancy | Non-diabetic | CTV3 |
| Q0143 | Fetus+tubal ectopic pregnancy | Non-diabetic | CTV3 |
| Q014z | Fetus+ectopic pregnancy NOS | Non-diabetic | CTV3 |
| Q015. | Fetus+multiple pregnancy | Non-diabetic | CTV3 |
| Q0150 | Fetus+multiple pregnancy unsp. | Non-diabetic | CTV3 |
| Q0151 | Fetus+twin pregnancy | Non-diabetic | CTV3 |
| Q0152 | Fetus+triplet pregnancy | Non-diabetic | CTV3 |
| Q015z | Fetus+multiple pregnancy NOS | Non-diabetic | CTV3 |
| ZV22. | [V]Normal pregnancy | Non-diabetic | CTV3 |
| ZV222 | [V]Pregnancy confirmed | Non-diabetic | CTV3 |
| ZV224 | [V]Supervis/oth norm pregnancy | Non-diabetic | CTV3 |
| ZV23. | [V]High-risk pregnancy superv. | Non-diabetic | CTV3 |
| ZV230 | [V]Pregnancy + PH infertility | Non-diabetic | CTV3 |
| ZV231 | [V]Pregnancy+PH trophobl.dis. | Non-diabetic | CTV3 |
| ZV232 | [V]Pregnancy + PH abortion | Non-diabetic | CTV3 |
| ZV23y | [V]OS high-risk pregnancy | Non-diabetic | CTV3 |
| ZV23z | [V]Unspec. high-risk pregnancy | Non-diabetic | CTV3 |
| L180900 | GDM | GDM | CTV3 |

## Table 2: Exclusion criteria codes

Note, as well as the codes below, anyone with a code suggestive of T2DM (code list present under the ‘Outcomes’ heading) at baseline was also excluded from the cohort

| **Diagnostic code** | **Description** | **Code type** |
| --- | --- | --- |
| C10E. | Type 1 diabetes mellitus | Read |
| C10EM | Type 1 diabetes mellitus with ketoacidosis | Read |
| C10E. | Type I diabetes mellitus | Read |
| C10E8 | Type 1 diabetes mellitus - poor control | Read |
| C10EE | Type 1 diabetes mellitus with hypoglycaemic coma | Read |
| C10E9 | Type 1 diabetes mellitus maturity onset | Read |
| C10EN | Type 1 diabetes mellitus with ketoacidotic coma | Read |
| C10E4 | Unstable type 1 diabetes mellitus | Read |
| C10E3 | Insulin dependent diabetes mellitus with multiple complicat | Read |
| C10E3 | Type 1 diabetes mellitus with multiple complications | Read |
| C10E4 | Unstable type I diabetes mellitus | Read |
| C10E. | Insulin dependent diabetes mellitus | Read |
| C10E4 | Unstable insulin dependent diabetes mellitus | Read |
| C10EM | Type I diabetes mellitus with ketoacidosis | Read |
| C10EN | Type I diabetes mellitus with ketoacidotic coma | Read |
| C10E8 | Insulin dependent diabetes mellitus - poor control | Read |
| C10E3 | Type I diabetes mellitus with multiple complications | Read |
| C10E9 | Type I diabetes mellitus maturity onset | Read |
| C10EE | Insulin dependent diabetes mellitus with hypoglycaemic coma | Read |
| C10E8 | Type I diabetes mellitus - poor control | ICD-10 |
| E10.0 | Insulin-dependant diabetes mellitus with coma | ICD-10 |
| E10.1 | Insulin-dependant diabetes mellitus with ketoacidosis | ICD-10 |
| E10.2 | Insulin-dependant diabetes mellitus with renal complications | ICD-10 |
| E10.3 | Insulin-dependant diabetes mellitus with ophthalmic complications | ICD-10 |
| E10.4 | Insulin-dependant diabetes mellitus with neurological complications | ICD-10 |
| E10.5 | Insulin-dependant diabetes mellitus with peripheral circulatory complications | ICD-10 |
| E10.6 | Insulin-dependant diabetes mellitus with other specified complications | ICD-10 |
| E10.7 | Insulin-dependant diabetes mellitus with multiple complications | ICD-10 |
| E10.8 | Insulin-dependant diabetes mellitus with unspecified complications | ICD-10 |
| E09.9 | Drug or chemical induced diabetes mellitus w/o complications | ICD-10 |
| E09.65 | Drug or chemical induced diabetes mellitus with hyperglycemia | ICD-10 |
| R10C. | [D]Drug induced hyperglycaemia | Read |
| C10H.00 | Diabetes mellitus induced by non-steroid drugs | Read |
| C10H000 | DM induced by non-steroid drugs without complication | Read |
| C10B.00 | Diabetes mellitus induced by steroids | Read |
| C10B000 | Steroid induced diabetes mellitus without complication | Read |

## Outcome codes

Table 3a: Type 2 Diabetes (note this code list was also used to detect and thus exclude subjects with T2DM prior to 2012)

| **Diagnostic code** | **Description** | **Code type** |
| --- | --- | --- |
| C1001 | Diab.mell.no comp. - adult | Read |
| C1031 | Type 2 diabetes mellitus with ketoacidotic coma | Read |
| C1051 | Diab.mell.+eye manif - adult | Read |
| C1061 | Diab.mell.+neuropathy - adult | Read |
| C1074 | NIDDM periph circulat disord | Read |
| C109. | Type 2 diabetes mellitus | Read |
| C1090 | Type 2 diabetes mellitus with renal complications | Read |
| C1091 | Type 2 diabetes mellitus with ophthalmic complications | Read |
| C1092 | Type 2 diabetes mellitus with neurological complications | Read |
| C1093 | Type 2 diabetes mellitus with multiple complications | Read |
| C1094 | Type 2 diabetes mellitus with ulcer | Read |
| C1095 | Type 2 diabetes mellitus with gangrene | Read |
| C1096 | Type 2 diabetes mellitus with retinopathy | Read |
| C1097 | Type 2 diabetes mellitus - poor control | Read |
| C1099 | Type II diabetes mellitus without complication | Read |
| C109A | Non-insulin dependent diabetes mellitus with mononeuropathy | Read |
| C109B | Non-insulin dependent diabetes mellitus with polyneuropathy | Read |
| C109C | Non inslulin dependant diab mell nephropathy | Read |
| C109D | Non-insulin dependent diabetes mellitus with hypoglycaemic coma | Read |
| C109E | Non-insulin dependent diabetes mellitus with diabetic cataract | Read |
| C109F | Non-insulin-dependent diabetes mellitus with peripheral angiopathy | Read |
| C109G | Non-insulin dependent diabetes mellitus with arthropathy | Read |
| C109H | Non-insulin dependent diabetes mellitus with neuropathic arthropathy | Read |
| C109J | Insulin treated Type 2 diabetes mellitus | Read |
| C109K | Hyperosmolar non-ketotic state in type 2 diabetes mellitus | Read |
| C10F. | Type 2 diabetes mellitus | Read |
| C10F0 | Type 2 diab mell + renal compl | Read |
| C10F1 | Type 2 diab mell+ophthal comp | Read |
| C10F2 | Type 2 diab mell + neurol comp | Read |
| C10F3 | Type 2 diabetes mellitus with multiple complications | Read |
| C10F4 | Type 2 diabetes mellitus with ulcer | Read |
| C10F5 | Type 2 diabetes mellitus with gangrene | Read |
| C10F6 | Type 2 diabetes mellitus with retinopathy | Read |
| C10F7 | Type 2 diabetes mellitus - poor control | Read |
| C10FA | Type 2 diabetes mellitus with mononeuropathy | Read |
| C10FB | Type 2 diab mell + polyneurop | Read |
| C10FC | Type 2 diabetes mellitus with nephropathy | Read |
| C10FD | Type 2 diabetes mellitus with hypoglycaemic coma | Read |
| C10FE | Type 2 diabetes mellitus with diabetic cataract | Read |
| C10FF | Type 2 diabetes mellitus with peripheral angiopathy | Read |
| C10FG | Type 2 diabetes mellitus with arthropathy | Read |
| C10FH | Type 2 diabetes mellitus with neuropathic arthropathy | Read |
| C10FJ | Insulin treated Type 2 diabetes mellitus | Read |
| C10FK | Hyperosmolar non-ketotic state in type 2 diabetes mellitus | Read |
| C10FL | Type 2 d m + persist proteinur | Read |
| C10FM | Type 2 d m + persist microalb | Read |
| C10FN | Type 2 diabetes mellitus with ketoacidosis | Read |
| C10FP | Type 2 diabetes mellitus with ketoacidotic coma | Read |
| C10FQ | Type 2 d m + exudat maculopath | Read |
| C10FR | Type 2 dm with gastroparesis | Read |
| L1806 | type 2 diabetes diagnosis | Read |
| C109.12 | Type 2 diabetes mellitus | Read |
| C109.13 | Type II diabetes mellitus | Read |
| C10F.11 | NIDDM - Non-insulin dependent diabetes mellitus | Read |
| C10F.00 | Type 2 diabetes mellitus | Read |
| C109.00 | Non-insulin dependent diabetes mellitus | Read |
| C109.11 | NIDDM - Non-insulin dependent diabetes mellitus | Read |
| C108900 | Insulin dependent diabetes maturity onset | Read |
| 66A4.00 | Diabetic on oral treatment | Read |
| C10FJ00 | Insulin treated Type 2 diabetes mellitus | Read |
| C109J12 | Insulin treated Type II diabetes mellitus | Read |
| C109J00 | Insulin treated Type 2 diabetes mellitus | Read |
| C109J11 | Insulin treated non-insulin dependent diabetes mellitus | Read |
| C109D11 | Type II diabetes mellitus with hypoglycaemic coma | Read |
| C109D12 | Type 2 diabetes mellitus with hypoglycaemic coma | Read |
| C10FD00 | Type 2 diabetes mellitus with hypoglycaemic coma | Read |
| C10FD11 | Type II diabetes mellitus with hypoglycaemic coma | Read |
| C109D00 | Non-insulin dependent diabetes mellitus with hypoglycaemic coma | Read |
| C10F711 | Type II diabetes mellitus - poor control | Read |
| C109712 | Type 2 diabetes mellitus - poor control | Read |
| C10F700 | Type 2 diabetes mellitus - poor control | Read |
| C109700 | Non-insulin dependent diabetes mellitus - poor control | Read |
| C109711 | Type II diabetes mellitus - poor control | Read |
| C10FK00 | Hyperosmolar non-ketotic state in type 2 diabetes mellitus | Read |
| C109K00 | Hyperosmolar non-ketotic state in type 2 diabetes mellitus | Read |
| C102100 | Diabetes mellitus, adult onset, with hyperosmolar coma | Read |
| C10FK11 | hyperosmolar non-ketotic state in type 2 diabetes mellitus | Read |
| C10FN00 | Type 2 diabetes mellitus with ketoacidosis | Read |
| C10FP00 | Type 2 diabetes mellitus with ketoacidotic coma | Read |
| C10FP11 | Type II diabetic mellitus with ketoacidotic coma | Read |
| C10FN11 | Type II diabetes mellitus with ketoacidosis | Read |
| E11.00 | Type 2 diabetes mellitus with hyperosmolarity without nonketotic hyperglycemic-hyperosmolar coma (NKHHC) | ICD-10 |
| E11.01 | Type 2 diabetes mellitus with hyperosmolarity with coma | ICD-10 |
| E11.65 | Type 2 diabetes mellitus with hyperglycemia | ICD-10 |
| E11.69 | Type 2 diabetes mellitus with other specified complication | ICD-10 |

Table 3b: Eye complication codes

| **Diagnostic code** | **Description** | **Code type** |
| --- | --- | --- |
| 2BBF. | Retina abnormal - diabet relat | Read |
| 2BBk. | O/E- R st treat prol diab ret | Read |
| 2BBL. | O/E - diabet maculop both eyes | Read |
| 2BBl. | O/E- L st treat prol diab ret | Read |
| 2BBM. | O/E - diab macul abs both eyes | Read |
| 2BBo. | O/E - sight threat diab retin | Read |
| 2BBP. | O/E - right eye back diab ret | Read |
| 2BBQ. | O/E - left eye back diab ret | Read |
| 2BBr. | Impair vision due diab retinop | Read |
| 2BBR. | O/E - R eye preprolif diab ret | Read |
| 2BBS. | O/E - L eye preprolif diab ret | Read |
| 2BBT. | O/E - R eye prolif diab ret | Read |
| 2BBV. | O/E - L eye prolif diab ret | Read |
| 2BBW. | O/E - R eye diab maculopathy | Read |
| 2BBX. | O/E - L eye diab maculopathy | Read |
| C109600 | Non-insulin-dependent diabetes mellitus with retinopathy | Read |
| C10F600 | Type 2 diabetes mellitus with retinopathy | Read |
| C10FQ00 | Type 2 diabetes mellitus with exudative maculopathy | Read |
| C109612 | Type 2 diabetes mellitus with retinopathy | Read |
| C10F611 | Type II diabetes mellitus with retinopathy | Read |
| C109611 | Type II diabetes mellitus with retinopathy | Read |
| C10F011 | Type II diabetes mellitus with retinopathy | Read |
| C1087 | Insulin dependent diabetes mellitus with retinopathy | Read |
| C10E7 | Type 1 diabetes mellitus with retinopathy | Read |
| C10EP | Type 1 diabetes mellitus with exudative maculopathy | Read |
| C1087 | Type I diabetes mellitus with retinopathy | Read |
| C1087 | Type 1 diabetes mellitus with retinopathy | Read |
| C10E7 | Insulin dependent diabetes mellitus with retinopathy | Read |
| C10E7 | Type I diabetes mellitus with retinopathy | Read |
| C10EP | Type I diabetes mellitus with exudative maculopathy | Read |
| E113 | Type II diabetes with ophthalmic complications | ICD10 |

Table 3c: End-stage Kidney Disease on Renal Replacement Therapy Codes

| **Diagnostic Code** | **Description** | **Code type** |
| --- | --- | --- |
| Z99.2 | Haemodialysis status and peritoneal dialysis status | ICD-10 |
| 7L1A200 | Haemodialysis NEC | Read |
| 761A2 | Haemodialysis | Read |
| 7L1A100 | Peritoneal dialysis | Read |
| 7L1A400 | Automated peritoneal dialysis | Read |
| 7L1A500 | Continuous ambulatory peritoneal dialysis | Read |
| 761A5 | Continuous ambulatory peritoneal dialysis | Read |
| 7L1A600 | Peritoneal dialysis NEC | Read |
| 761a1 | Peritoneal dialysis | Read |
| 7L1A.11 | Dialysis for renal failure | Read |
| 761A0 | Renal dialysis | Read |
| 761A5 | Continuous Ambulatory Peritoneal Dialysis | Read |
| X402 | Peritoneal dialysis NEC | OPCS |
| X403 | Haemodialysis NEC | OPCS |
| X405 | Automated peritoneal dialysis | OPCS |
| X406 | Continuous ambulatory peritoneal dialysis | OPCS |
| 7B00 | Transplantation of the kidney | Read |
| 7B001 | Transplantation of the kidney from a live donor | Read |
| 7B00200 | Transplantation of kidney from Cadaver | Read |
| 7B00211 | Allotransplantation from cadaver | Read |
| 7B00212 | Cadaveric renal transplant | Read |
| 7B00300 | Allotransplantation from cadaver, heart beating | Read |
| 7B00400 | Allotransplantation kidney from cadaver, heart non-beating | Read |
| 7B012 | Bilateral nephrectomy | Read |
| ZV420 | Kidney transplanted | ICD10 |
| 7B015 | Transplant nephrectomy | Read |
| 7B019 | Excision of rejected transplant kidney | Read |
| 7B063 | Exploration of renal transplant | Read |
| 7B0F3 | Pos-transplantation of Kidney examination, recipient | Read |
| 8l50.00 | Renal transplant planned | Read |
| E13.019 | Simultaneous Pancreas and kidney transplant | ICD10 |
| M013 | Allotransplant of the kidney from cadaver NEC | OPCS |
| M014 | Allotransplantation of kidney from cadaver heart beating | OPCS |
| M018 | Other specified transplantation of kidney | OPCS |

Table 3d: Cataract

| **Diagnostic code** | **Description** | **Code type** |
| --- | --- | --- |
| C109E12 | Type 2 diabetes mellitus with diabetic cataract | Read |
| C10FE00 | Type 2 diabetes mellitus with diabetic cataract | Read |
| C109E11 | Type II diabetes mellitus with diabetic cataract | Read |
| C10FE11 | Type II diabetes mellitus with diabetic cataract | Read |
| C10EF | Type 1 diabetes mellitus with diabetic cataract | Read |
| C10EF | Insulin dependent diabetes mellitus with diabetic cataract | Read |
| C108F | Insulin dependent diabetes mellitus with diabetic cataract | Read |
| C109E00 | non-insulin dependent diabetes mellitus with diabetic cataract | Read |
| C108F | Type I diabetes mellitus with diabetic cataract | Read |

Table 3e: Ischaemic heart disease

| **Diagnostic code** | **Description** | **Code type** |
| --- | --- | --- |
| K751 | Percutaneous transluminal balloon angioplasty and insertion of 1-2 drug-eluting stents into coronary artery | OPCS |
| K752 | Percutaneous transluminal balloon angioplasty and insertion of 3 or more drug-eluting stents into coronary artery | OPCS |
| K753 | Percutaneous transluminal balloon angioplasty and insertion of 1-2 stents into coronary artery | OPCS |
| K754 | Percutaneous transluminal balloon angioplasty and insertion of 3 or more stents into coronary artery NEC | OPCS |
| K758 | Other specified percutaneous transluminal balloon angioplasty and insertion of stent into coronary artery | OPCS |
| K759 | Unspecified percutaneous transluminal balloon angioplasty and insertion of stent into coronary artery | OPCS |
| K491 | Percutaneous transluminal balloon angioplasty of one coronary artery | OPCS |
| K492 | Percutaneous transluminal balloon angioplasty of multiple coronary arteries | OPCS |
| K493 | Percutaneous transluminal balloon angioplasty of bypass graft of coronary artery | OPCS |
| K494 | Percutaneous transluminal cutting balloon angioplasty of coronary artery | OPCS |
| K498 | Other specified transluminal balloon angioplasty of coronary artery | OPCS |
| K499 | Unspecified transluminal balloon angioplasty of coronary artery | OPCS |
| K501 | Percutaneous transluminal laser coronary angioplasty | OPCS |
| K502 | Percutaneous transluminal coronary thrombolysis using streptokinase | OPCS |
| K503 | Percutaneous transluminal injection of therapeutic substance into coronary artery NEC | OPCS |
| K504 | Percutaneous transluminal atherectomy of coronary artery | OPCS |
| K508 | Other specified other therapeutic transluminal operations on coronary artery | OPCS |
| K509 | Unspecified other therapeutic transluminal operations on coronary artery | OPCS |
| K401 | Saphenous vein graft replacement of one coronary artery | OPCS |
| K402 | Saphenous vein graft replacement of two coronary arteries | OPCS |
| K403 | Saphenous vein graft replacement of three coronary arteries | OPCS |
| K404 | Saphenous vein graft replacement of four or more coronary arteries | OPCS |
| K409 | Unspecified saphenous vein graft replacement of coronary artery | OPCS |
| K411 | Autograft replacement of one coronary artery NEC | OPCS |
| K412 | Autograft replacement of two coronary arteries NEC | OPCS |
| K413 | Autograft replacement of three coronary arteries NEC | OPCS |
| K414 | Autograft replacement of four or more coronary arteries NEC | OPCS |
| K419 | Unspecified other autograft replacement of coronary artery | OPCS |
| K421 | Allograft replacement of one coronary artery | OPCS |
| K422 | Allograft replacement of two coronary arteries | OPCS |
| K423 | Allograft replacement of three coronary arteries | OPCS |
| K429 | Unspecified allograft replacement of coronary artery | OPCS |
| K431 | Prosthetic replacement of one coronary artery | OPCS |
| K432 | Prosthetic replacement of two coronary arteries | OPCS |
| K433 | Prosthetic replacement of three coronary arteries | OPCS |
| K434 | Prosthetic replacement of four or more coronary arteries | OPCS |
| K439 | Unspecified prosthetic replacement of coronary artery | OPCS |
| K441 | Replacement of coronary arteries using multiple methods | OPCS |
| K442 | Revision of replacement of coronary artery | OPCS |
| K448 | Other specified other replacement of coronary artery | OPCS |
| K449 | Unspecified other replacement of coronary artery | OPCS |
| K451 | Double anastomosis of mammary arteries to coronary arteries | OPCS |
| K452 | Double anastomosis of thoracic arteries to coronary arteries NEC | OPCS |
| K453 | Anastomosis of mammary artery to left anterior descending coronary artery | OPCS |
| K454 | Anastomosis of mammary artery to coronary artery NEC | OPCS |
| K455 | Anastomosis of thoracic artery to coronary artery NEC | OPCS |
| K456 | Revision of connection of thoracic artery to coronary artery | OPCS |
| K458 | Other specified connection of thoracic artery to coronary artery | OPCS |
| K459 | Unspecified connection of thoracic artery to coronary artery | OPCS |
| K461 | Double implantation of mammary arteries into heart | OPCS |
| K463 | Implantation of mammary artery into heart NEC | OPCS |
| K468 | Other specified other bypass of coronary artery | OPCS |
| K469 | Unspecified other bypass of coronary artery | OPCS |
| I200 | Unstable angina | ICD10 |
| I201 | Angina pectoris with documented spasm | ICD10 |
| I208 | Other forms of angina pectoris | ICD10 |
| I209 | Angina pectoris, unspecified | ICD10 |
| I210 | Acute transmural myocardial infarction of anterior wall | ICD10 |
| I211 | Acute transmural myocardial infarction of inferior wall | ICD10 |
| I212 | Acute transmural myocardial infarction of other sites | ICD10 |
| I213 | Acute transmural myocardial infarction of unspecified site | ICD10 |
| I214 | Acute subendocardial myocardial infarction | ICD10 |
| I219 | Acute myocardial infarction, unspecified | ICD10 |
| I220 | Subsequent myocardial infarction of anterior wall | ICD10 |
| I221 | Subsequent myocardial infarction of inferior wall | ICD10 |
| I228 | Subsequent myocardial infarction of other sites | ICD10 |
| I229 | Subsequent myocardial infarction of unspecified site | ICD10 |
| I230 | Haemopericardium as current complication following acute myocardial infarction | ICD10 |
| I231 | Atrial septal defect as current complication following acute myocardial infarction | ICD10 |
| I232 | Ventricular septal defect as current complication following acute myocardial infarction | ICD10 |
| I233 | Rupture of cardiac wall without haemopericardium as current complication following acute myocardial infarction | ICD10 |
| I234 | Rupture of chordae tendineae as current complication following acute myocardial infarction | ICD10 |
| I235 | Rupture of papillary muscle as current complication following acute myocardial infarction | ICD10 |
| I236 | Thrombosis of atrium, auricular appendage, and ventricle as current complications following acute myocardial infarction | ICD10 |
| I238 | Other current complications following acute myocardial infarction | ICD10 |
| I240 | Coronary thrombosis not resulting in myocardial infarction | ICD10 |
| I248 | Other forms of acute ischaemic heart disease | ICD10 |
| I249 | Acute ischaemic heart disease, unspecified | ICD10 |
| I250 | Atherosclerotic cardiovascular disease, so described | ICD10 |
| I251 | Atherosclerotic heart disease | ICD10 |
| I252 | Old myocardial infarction | ICD10 |
| I254 | Coronary artery aneurysm and dissection | ICD10 |
| I255 | Ischaemic cardiomyopathy | ICD10 |
| I256 | Silent myocardial ischaemia | ICD10 |
| I258 | Other forms of chronic ischaemic heart disease | ICD10 |
| I259 | Chronic ischaemic heart disease, unspecified | ICD10 |
| G3... | Ischaemic heart disease | Read |
| G30.. | Acute myocardial infarction | Read |
| G300. | Acute anterolateral infarction | Read |
| G301. | Anterior myocard. infarct OS | Read |
| G3010 | Acute anteroapical infarction | Read |
| G3011 | Acute anteroseptal infarction | Read |
| G301z | Anterior myocard.infarct NOS | Read |
| G302. | Acute inferolateral infarction | Read |
| G303. | Acute inferoposterior infarct | Read |
| G304. | Posterior myocard.infarct NOS | Read |
| G305. | Lateral myocardial infarct NOS | Read |
| G306. | True posterior myocard.infarct | Read |
| G307. | Acute subendocardial infarct | Read |
| G3070 | Acute non-Q wave infarction | Read |
| G3071 | Acute non-ST seg elevation mi | Read |
| G308. | Inferior myocard. infarct NOS | Read |
| G309. | Acute Q-wave infarct | Read |
| G30B. | Acute posterol myocard infarct | Read |
| G30X. | Ac transmur MI unspec site | Read |
| G30X0 | Acute ST segment elevation mi | Read |
| G30y. | Other acute myocardial infarct | Read |
| G30y0 | Acute atrial infarction | Read |
| G30y1 | Acute papillary muscle infarct | Read |
| G30y2 | Acute septal infarction | Read |
| G30yz | Other acute myocardial inf.NOS | Read |
| G30z. | Acute myocardial infarct. NOS | Read |
| G31.. | Other acute/subacute IHD | Read |
| G311. | Preinfarction syndrome | Read |
| G3110 | Myocardial infarction aborted | Read |
| G3111 | Unstable angina | Read |
| G3112 | Angina at rest | Read |
| G3113 | Refractory angina | Read |
| G3114 | Worsening angina | Read |
| G3115 | Acute coronary syndrome | Read |
| G311z | Preinfarction syndrome NOS | Read |
| G312. | Coron thromb/not result in MI | Read |
| G31y. | Other acute/subacute IHD | Read |
| G31y0 | Acute coronary insufficiency | Read |
| G31y1 | Microinfarction of heart | Read |
| G31y2 | Subendocardial ischaemia | Read |
| G31y3 | Transient myocardial ischaemia | Read |
| G31yz | Other acute/subacute IHD NOS | Read |
| G32.. | Old myocardial infarction | Read |
| G33.. | Angina pectoris | Read |
| G330. | Angina decubitus | Read |
| G3300 | Nocturnal angina | Read |
| G330z | Angina decubitus NOS | Read |
| G33z. | Angina pectoris NOS | Read |
| G33z0 | Status anginosus | Read |
| G33z1 | Stenocardia | Read |
| G33z2 | Syncope anginosa | Read |
| G33z3 | Angina on effort | Read |
| G33z4 | Ischaemic chest pain | Read |
| G33z5 | Post infarct angina | Read |
| G33z6 | New onset angina | Read |
| G33z7 | Stable angina | Read |
| G33zz | Angina pectoris NOS | Read |
| G34.. | Other chr.ischaemic heart dis. | Read |
| G340. | Coronary atherosclerosis | Read |
| G3400 | Single coronary vessel disease | Read |
| G3401 | Double coronary vessel disease | Read |
| G342. | Atherosclerotic cardiovasc dis | Read |
| G343. | Ischaemic cardiomyopathy | Read |
| G344. | Silent myocardial ischaemia | Read |
| G34y. | Other specif. chronic IHD | Read |
| G34y0 | Chronic coronary insufficiency | Read |
| G34y1 | Chronic myocardial ischaemia | Read |
| G34yz | Other specif.chronic IHD NOS | Read |
| G34z. | Other chronic IHD NOS | Read |
| G34z0 | Asymptomatic CHD | Read |
| G35.. | Subseqnt myocardial infarction | Read |
| G350. | Subsqnt myocrd infarc/ant wall | Read |
| G351. | Subsqnt myocrd infarc/inf wall | Read |
| G353. | Subseq myo infarct other sites | Read |
| G35X. | Subseq MI of unspec site | Read |
| G38.. | Postoperative MI | Read |
| G380. | Postop transmur MI inf wall | Read |
| G381. | Postop transm MI inferior wall | Read |
| G382. | Postop transm MI other sites | Read |
| G383. | Postop transm MI unspec site | Read |
| G384. | Postop subendocardial MI | Read |
| G38z. | Postop MI,unspec | Read |
| G39.. | Coronary microvascular disease | Read |
| G3y.. | Ischaemic heart disease OS | Read |
| G3z.. | Ischaemic heart disease NOS | Read |

Table 3f: Heart failure

| **Diagnostic code** | **Description** | **Code type** |
| --- | --- | --- |
| I500 | Congestive heart failure | ICD10 |
| I501 | Left ventricular failure | ICD10 |
| I110 | Hypertensive heart disease with (congestive) heart failure | ICD10 |
| I130 | Hypertensive heart and renal disease with (congestive) heart failure | ICD10 |
| I132 | Hypertensive heart and renal disease with both (congestive) heart failure and renal failure | ICD10 |
| 662F. | NYHA classification - class I | Read |
| 662G. | NYHA classification - class II | Read |
| 662H. | NYHA classification- class III | Read |
| 662I. | NYHA classification - class IV | Read |
| G1yz1 | Rheumatic left ventric.failure | Read |
| G58.. | Heart failure | Read |
| G580. | Congestive heart failure | Read |
| G5800 | Acute congestive heart failure | Read |
| G5801 | Chroncongestive heart failure | Read |
| G5802 | Decompensated cardiac failure | Read |
| G5803 | Compensated cardiac failure | Read |
| G5804 | Cong heart fail due valv dis | Read |
| G581. | Left ventricular failure | Read |
| G5810 | Acute left ventricular failure | Read |
| G582. | Acute heart failure | Read |
| G583. | Heart failure norm eject frac | Read |
| G584. | Right ventricular failure | Read |
| G58z. | Heart failure NOS | Read |

Table 3g: Vascular disease

| **Diagnostic Code** | **Description** | **Code Type** |
| --- | --- | --- |
| 1M110 | Ischaemic foot pain at rest-yes | Read |
| 1M111 | Ischaemic foot pain when walking-yes | Read |
| 24EA. | O/E: Absent right foot pulses-yes | Read |
| 24FA. | O/E: Absent left foot pulses-yes | Read |
| 1M110 | Ischaemic foot pain at rest-yes | Read |
| 1M111 | Ischaemic foot pain when walking-yes | Read |
| 24EA. | O/E: Absent right foot pulses-yes | Read |
| 24FA. | O/E: Absent left foot pulses-yes | Read |
| I700 | Atherosclerosis of aorta | ICD10 |
| I701 | Atherosclerosis of renal artery | ICD10 |
| I702 | Atherosclerosis of arteries of extremities | ICD10 |
| I708 | Atherosclerosis of other arteries | ICD10 |
| I709 | Generalized and unspecified atherosclerosis | ICD10 |
| I710 | Dissection of aorta [any part] | ICD10 |
| I711 | Thoracic aortic aneurysm, ruptured | ICD10 |
| I712 | Thoracic aortic aneurysm, without mention of rupture | ICD10 |
| I713 | Abdominal aortic aneurysm, ruptured | ICD10 |
| I714 | Abdominal aortic aneurysm, without mention of rupture | ICD10 |
| I715 | Thoracoabdominal aortic aneurysm, ruptured | ICD10 |
| I716 | Thoracoabdominal aortic aneurysm, without mention of rupture | ICD10 |
| I718 | Aortic aneurysm of unspecified site, ruptured | ICD10 |
| I719 | Aortic aneurysm of unspecified site, without mention of rupture | ICD10 |
| I720 | Aneurysm and dissection of carotid artery | ICD10 |
| I721 | Aneurysm and dissection of artery of upper extremity | ICD10 |
| I722 | Aneurysm and dissection of renal artery | ICD10 |
| I723 | Aneurysm and dissection of iliac artery | ICD10 |
| I724 | Aneurysm and dissection of artery of lower extremity | ICD10 |
| I728 | Aneurysm and dissection of other specified arteries | ICD10 |
| I729 | Aneurysm and dissection of unspecified site | ICD10 |
| I738 | Other specified peripheral vascular diseases | ICD10 |
| I739 | Peripheral vascular disease, unspecified | ICD10 |
| I740 | Embolism and thrombosis of abdominal aorta | ICD10 |
| I741 | Embolism and thrombosis of other and unspecified parts of aorta | ICD10 |
| I742 | Embolism and thrombosis of arteries of upper extremities | ICD10 |
| I743 | Embolism and thrombosis of arteries of lower extremities | ICD10 |
| I744 | Embolism and thrombosis of arteries of extremities, unspecified | ICD10 |
| I745 | Embolism and thrombosis of iliac artery | ICD10 |
| I748 | Embolism and thrombosis of other arteries | ICD10 |
| I749 | Embolism and thrombosis of unspecified artery | ICD10 |
| I771 | Stricture of artery | ICD10 |
| I772 | Rupture of artery | ICD10 |
| I778 | Other specified disorders of arteries and arterioles | ICD10 |
| I779 | Disorder of arteries and arterioles, unspecified | ICD10 |
| I790 | Aneurysm of aorta in diseases classified elsewhere | ICD10 |
| I7000 | Atherosclerosis of aorta | ICD10 |
| I7001 | Atherosclerosis of aorta | ICD10 |
| I7010 | Atherosclerosis of renal artery | ICD10 |
| I7011 | Atherosclerosis of renal artery | ICD10 |
| I7020 | Atherosclerosis of arteries of extremities | ICD10 |
| I7021 | Atherosclerosis of arteries of extremities | ICD10 |
| I7091 | Generalized and unspecified atherosclerosis | ICD10 |
| I725 | Aneurysm and dissection of other precerebral arteries | ICD10 |
| I726 | Aneurysm and dissection of vertebral artery | ICD10 |
| E125 | With peripheral circulatory complications | ICD10 |
| E145 | With peripheral circulatory complications | ICD10 |
| E135 | With peripheral circulatory complications | ICD10 |
| E115 | With peripheral circulatory complications | ICD10 |
| C10799 | Diabetes + periph.circulat.dis | Read |
| C107z | Diabetes mellitus NOS with peripheral circulatory disorder | Read |
| C1071 | Diabetes mellitus, adult, + peripheral circulatory disorder | Read |
| C1074 | NIDDM with peripheral circulatory disorder | Read |
| C109F | Non-insulin-dependent d m with peripheral angiopath | Read |
| C107. | Diabetes mellitus with peripheral circulatory disorder | Read |
| C107y | Other specified diabetes mellitus with periph circ comps | Read |
| C10FF | Type 2 diabetes mellitus with peripheral angiopathy | Read |
| C109F2 | Type 2 diabetes mellitus with peripheral angiopathy | Read |
| C10FF | Type 2 diabetes mellitus with peripheral angiopathy | Read |
| C10FF | Type 2 diabetes mellitus with peripheral angiopathy | Read |
| C109F2 | Type 2 diabetes mellitus with peripheral angiopathy | Read |
| C109F2 | Type 2 diabetes mellitus with peripheral angiopathy | Read |
| C10FF1 | Type II diabetes mellitus with peripheral angiopathy | Read |
| C109F1 | Type II diabetes mellitus with peripheral angiopathy | Read |
| C109F1 | Type II diabetes mellitus with peripheral angiopathy | Read |
| C10FF1 | Type II diabetes mellitus with peripheral angiopathy | Read |
| C109F1 | Type II diabetes mellitus with peripheral angiopathy | Read |
| C10FF00 | Type 2 diabetes mellitus with peripheral angiopathy | Read |
| C109F11 | Type II diabetes mellitus with peripheral angiopathy | Read |
| C109F12 | Type 2 diabetes mellitus with peripheral angiopathy | Read |
| C107400 | NIDDM with peripheral circulatory disorder | Read |
| C107100 | Diabetes mellitus, adult, + peripheral circulatory disorder | Read |
| C10F511 | Type 2 diabetes mellitus with peripheral angiopathy | Read |
| C10FF11 | Type 2 diabetes mellitus with peripheral angiopathy | Read |
| G73.. | Other peripheral vascular dis. | Read |
| G734. | Peripheral arterial disease | Read |
| G73y. | Other spec.periph.vasc.disease | Read |
| G73z. | Peripheral vascular dis. NOS | Read |
| G73z0 | Intermittent claudication | Read |
| G73zz | Peripheral vasc.disease NOS | Read |
| Gyu74 | [X]Oth spcf periph vasculr dis | Read |
| C108G | Insulin dependent diab mell with peripheral angiopathy | Read |

Table 3h: Neuropathy

| **Diagnostic Code** | **Description** | **Code type** |
| --- | --- | --- |
| C10699 | Diabetes + neuropathy | Read |
| F3722 | Asymptomatic diabetic neuropathy | Read |
| C106z | Diabetes mellitus NOS with neurological manifestation | Read |
| C106y | Other specified diabetes mellitus with neurological comps | Read |
| C1061 | Diabetes mellitus, adult onset, + neurological manifestation | Read |
| F3y0. | Diabetic mononeuropathy | Read |
| C1062 | Diabetes mellitus with neuropathy | Read |
| C10FB1 | Type II diabetes mellitus with polyneuropathy | Read |
| C109B1 | Type II diabetes mellitus with polyneuropathy | Read |
| C1092 | Non-insulin-dependent diabetes mellitus with neuro comps | Read |
| C10F21 | Type II diabetes mellitus with neurological complications | Read |
| C10921 | Type II diabetes mellitus with neurological complications | Read |
| C10FA | Type 2 diabetes mellitus with mononeuropathy | Read |
| C109A2 | Type 2 diabetes mellitus with mononeuropathy | Read |
| F372. | Polyneuropathy in diabetes | Read |
| C10922 | Type 2 diabetes mellitus with neurological complications | Read |
| C10F2 | Type 2 diabetes mellitus with neurological complications | Read |
| C10FA1 | Type II diabetes mellitus with mononeuropathy | Read |
| C109A1 | Type II diabetes mellitus with mononeuropathy | Read |
| C106y00 | Other specified diabetes mellitus with neurological comps | Read |
| C10FB00 | Type 2 diabetes mellitus with polyneuropathy | Read |
| C10F200 | Type 2 diabetes mellitus with neurological complications | Read |
| C106100 | Diabetes mellitus, adult onset, + neurological manifestation | Read |
| C109B00 | Non-insulin dependent diabetes mellitus with polyneuropathy | Read |
| C109212 | Type 2 diabetes mellitus with neurological complications | Read |
| C10FB11 | Type II diabetes mellitus with polyneuropathy | Read |
| C109A11 | Type II diabetes mellitus with mononeuropathy | Read |
| C10FA00 | Type 2 diabetes mellitus with mononeuropathy | Read |
| C10FA11 | type 2 diabetes mellitus with mononeuropathy | Read |
| C10F211 | Type 2 diabetes mellitus with neurological complications | Read |
| C109211 | Type 2 diabetes mellitus with neurological complications | Read |
| C109A00 | non-insulin dependent diabetes mellitus with mononeuropathy | Read |
| C109B11 | Type II diabetes mellitus with polyneuropathy | Read |
| F3721 | Chronic painful diabetic neuropathy | Read |
| G73y0 | Diabetic peripheral angiopathy | Read |
| C109B2 | Type 2 diabetes mellitus with polyneuropathy | Read |
| F3720 | Acute painful diabetic neuropathy | Read |
| F1711 | Autonomic neuropathy due to diabetes | Read |
| C109200 | Non-insulin-dependent diabetes mellitus with neuro comps | Read |
| F3722 | Diabetic neuropathy | Read |
| 1M8.. | Diabetic peripheral neuropathic pain | Read |
| C109A | Non-insulin dependent diabetes mellitus with mononeuropathy | Read |
| C109B | Non-insulin dependent diabetes mellitus with polyneuropathy | Read |
| F3720 | Acute painful diab neuropathy | Read |
| F3721 | Chron painful diab neuropathy | Read |
| C109G11 | Type II diabetes mellitus with arthropathy | Read |
| C109H11 | Type II diabetes mellitus with neuropathic arthropathy | Read |
| C10FG | Type 2 diabetes mellitus with arthropathy | Read |
| C109G2 | Type 2 diabetes mellitus with arthropathy | Read |
| C10FH1 | Type II diabetes mellitus with neuropathic arthropathy | Read |
| C109H1 | Type II diabetes mellitus with neuropathic arthropathy | Read |
| C109G1 | Type II diabetes mellitus with arthropathy | Read |
| C10FH00 | Type 2 diabetes mellitus with neuropathic arthropathy | Read |
| C109G12 | Type 2 diabetes mellitus with arthropathy | Read |
| C10FG11 | Type 2 diabetes mellitus with arthropathy | Read |
| C10FG1 | Type II diabetes mellitus with arthropathy | Read |
| C10FH | Type 2 diabetes mellitus with neuropathic arthropathy | Read |
| C109H2 | Type 2 diabetes mellitus with neuropathic arthropathy | Read |
| C109G00 | Non-insulin dependent diabetes mellitus with arthropathy | Read |
| C109H00 | Non-insulin dependent d m with neuropathic arthropathy | Read |
| C10FG00 | Type 2 diabetes mellitus with arthropathy | Read |
| C10FR00 | Type 2 diabetes mellitus with gastroparesis | Read |
| C108J | Type 1 diabetes mellitus with neuropathic arthropathy | Read |
| C10EH | Type 1 diabetes mellitus with arthropathy | Read |
| C108B | Insulin dependent diabetes mellitus with mononeuropathy | Read |
| C108J | Insulin dependent diab mell with neuropathic arthropathy | Read |
| C10E2 | Type 1 diabetes mellitus with neurological complications | Read |
| C10EC | Type 1 diabetes mellitus with polyneuropathy | Read |
| C1082 | Type I diabetes mellitus with neurological complications | Read |
| C1082 | Insulin-dependent diabetes mellitus with neurological comps | Read |
| C10EJ | Type 1 diabetes mellitus with neuropathic arthropathy | Read |
| C10EQ | Type 1 diabetes mellitus with gastroparesis | Read |
| C108J | Type I diabetes mellitus with neuropathic arthropathy | Read |
| C1082 | Type 1 diabetes mellitus with neurological complications | Read |
| C108H | Type I diabetes mellitus with arthropathy | Read |
| C108H | Insulin dependent diabetes mellitus with arthropathy | Read |
| C1060 | Diabetes mellitus, juvenile, + neurological manifestation | Read |
| C10EB | Type 1 diabetes mellitus with mononeuropathy | Read |
| C10EC | Type I diabetes mellitus with polyneuropathy | Read |
| C108B | Type I diabetes mellitus with mononeuropathy | Read |
| C10EC | Insulin dependent diabetes mellitus with polyneuropathy | Read |
| C10E2 | Insulin-dependent diabetes mellitus with neurological comps | Read |
| C10EQ | Type I diabetes mellitus with gastroparesis | Read |

Table 3i: Diabetic foot disease

| **Diagnostic code** | **Description** | **Code type** |
| --- | --- | --- |
| R0542 | [D]Gangrene of toe in diabetic | Read |
| R0543 | [D]Widespread diabetic foot gangrene | Read |
| 2G510 | Foot abnormal-diabetes related | Read |
| 2G5C. | Foot abnormality - diabetes related | Read |
| M2710 | Ischaemic ulcer diabetic foot | Read |
| M2712 | Mixed diabetic ulcer - foot | Read |
| M2714 | Neurogenic leg ulcer | Read |
| M2711 | Neuropathic diabetic ulcer - foot | Read |
| M2717 | Neuropathic foot ulcer | Read |
| 2G5W. | O/E - L chron diab foot ulcer | Read |
| 2G5L. | O/E - L diab foot - ulcerated | Read |
| 2G5W | O/E - left chronic diabetic foot ulcer | Read |
| 2G5L. | O/E - Left diabetic foot - ulcerated | Read |
| 2G5V. | O/E - R chron diab foot ulcer | Read |
| 2G5H. | O/E - R diab foot - ulcerated | Read |
| 2G5V. | O/E - right chronic diabetic foot ulcer | Read |
| 2G5H. | O/E - Right diabetic foot - ulcerated | Read |
| 2G4E. | Healred leg ulcer | Read |
| M0372 | Cellulitis in diabetic foot | Read |
| 2G510 | Foot abnormality - diabetes related-yes | Read |
| 2G5C. | Foot abnormality - diabetes related-yes | Read |
| 2G5H. | O/E - Right diabetic foot – ulcerated-yes | Read |
| 2G5L. | O/E - Left diabetic foot – ulcerated-yes | Read |
| 2G5W. | O/E - left chronic diabetic foot ulcer-yes | Read |
| M2710 | Ischaemic ulcer diabetic foot | Read |
| M2712 | Mixed diabetic ulcer - foot | Read |
| M2714 | Neurogenic leg ulcer | Read |
| M2711 | Neuropathic diabetic ulcer - foot | Read |
| M2717 | Neuropathic foot ulcer | Read |
| 2G5W. | O/E - L chron diab foot ulcer | Read |
| 2G5L. | O/E - L diab foot - ulcerated | Read |
| 2G5W | O/E - left chronic diabetic foot ulcer | Read |
| 2G5L. | O/E - Left diabetic foot - ulcerated | Read |
| 2G5V. | O/E - R chron diab foot ulcer | Read |
| 2G5H. | O/E - R diab foot - ulcerated | Read |
| 2G5V. | O/E - right chronic diabetic foot ulcer | Read |
| 2G5H. | O/E - Right diabetic foot - ulcerated | Read |
| 2G510 | Foot abnormal-diabetes related | Read |
| 2G5C. | Foot abnormal-diabetes related | Read |
| 2G510 | Foot abnormality - diabetes related | Read |
| 2G5C. | Foot abnormality - diabetes related | Read |
| R0542 | [D]Gangrene of toe in diabetic | Read |
| R0543 | [D]Widespread diabetic foot gangrene | Read |
| 2G510 | Foot abnormality - diabetes related-yes | Read |
| 2G5C. | Foot abnormality - diabetes related-yes | Read |
| 2G5H. | O/E - Right diabetic foot – ulcerated-yes | Read |
| 2G5L. | O/E - Left diabetic foot – ulcerated-yes | Read |
| 2G5W. | O/E - left chronic diabetic foot ulcer-yes | diabetic foot |

Table 3j: Amputation

| **Diagnostic Code** | **Description** | **Code type** |
| --- | --- | --- |
| X093 | Amputation of leg above knee | OPCS |
| X094 | Amputation of leg through knee | OPCS |
| X095 | Amputation of leg below knee | OPCS |
| X098 | Other specified amputation of leg | OPCS |
| X099 | Unspecified amputation of leg | OPCS |
| X101 | Amputation of foot through ankle | OPCS |
| X102 | Disarticulation of tarsal bones | OPCS |
| X103 | Disarticulation of metatarsal bones | OPCS |
| X104 | Amputation through metatarsal bones | OPCS |
| X108 | Other specified amputation of foot | OPCS |
| X109 | Unspecified amputation of foot | OPCS |
| X111 | Amputation of great toe | OPCS |
| X112 | Amputation of phalanx of toe | OPCS |
| X118 | Other specified amputation of toe | OPCS |
| X119 | Unspecified amputation of toe | OPCS |
| X093 | Amputation of leg above knee | OPCS |
| X094 | Amputation of leg through knee | OPCS |
| X095 | Amputation of leg below knee | OPCS |
| X098 | Other specified amputation of leg | OPCS |
| X099 | Unspecified amputation of leg | OPCS |
| X101 | Amputation of foot through ankle | OPCS |
| X102 | Disarticulation of tarsal bones | OPCS |
| X103 | Disarticulation of metatarsal bones | OPCS |
| X104 | Amputation through metatarsal bones | OPCS |
| X108 | Other specified amputation of foot | OPCS |
| X109 | Unspecified amputation of foot | OPCS |
| X111 | Amputation of great toe | OPCS |
| X112 | Amputation of phalanx of toe | OPCS |
| X118 | Other specified amputation of toe | OPCS |
| X119 | Unspecified amputation of toe | OPCS |

Table 3k: Cerebrovascular disease - ischaemic

| **Diagnostic code** | **Description** | **Code type** |
| --- | --- | --- |
| I630 | Cerebral infarction due to thrombosis of precerebral arteries | ICD10 |
| I631 | Cerebral infarction due to embolism of precerebral arteries | ICD10 |
| I632 | Cerebral infarction due to unspecified occlusion or stenosis of precerebral arteries | ICD10 |
| I633 | Cerebral infarction due to thrombosis of cerebral arteries | ICD10 |
| I634 | Cerebral infarction due to embolism of cerebral arteries | ICD10 |
| I635 | Cerebral infarction due to unspecified occlusion or stenosis of cerebral arteries | ICD10 |
| I636 | Cerebral infarction due to cerebral venous thrombosis, nonpyogenic | ICD10 |
| I638 | Other cerebral infarction | ICD10 |
| I639 | Cerebral infarction, unspecified | ICD10 |
| I650 | Occlusion and stenosis of vertebral artery | ICD10 |
| I651 | Occlusion and stenosis of basilar artery | ICD10 |
| I652 | Occlusion and stenosis of carotid artery | ICD10 |
| I653 | Occlusion and stenosis of multiple and bilateral precerebral arteries | ICD10 |
| I658 | Occlusion and stenosis of other precerebral artery | ICD10 |
| I659 | Occlusion and stenosis of unspecified precerebral artery | ICD10 |
| I660 | Occlusion and stenosis of middle cerebral artery | ICD10 |
| I661 | Occlusion and stenosis of anterior cerebral artery | ICD10 |
| I662 | Occlusion and stenosis of posterior cerebral artery | ICD10 |
| I663 | Occlusion and stenosis of cerebellar arteries | ICD10 |
| I664 | Occlusion and stenosis of multiple and bilateral cerebral arteries | ICD10 |
| I668 | Occlusion and stenosis of other cerebral artery | ICD10 |
| I669 | Occlusion and stenosis of unspecified cerebral artery | ICD10 |
| I693 | Sequelae of cerebral infarction | ICD10 |
| G63y0 | Cerebr infct/throm/precere art | Read |
| G63y1 | Cerebr infct/embol/precere art | Read |
| G64.. | Cerebral arterial occlusion | Read |
| G640. | Cerebral thrombosis | Read |
| G6400 | Cerebr infct/throm/cerebrl art | Read |
| G641. | Cerebral embolism | Read |
| G6410 | Cerebr infct/embol/cerebrl art | Read |
| G64z. | Cerebral infarction NOS | Read |
| G64z0 | Brainstem infarction | Read |
| G64z1 | Wallenberg syndrome | Read |
| G64z2 | Left sided cerebral infarction | Read |
| G64z3 | Right sided cerebral infarct | Read |
| G64z4 | Infarction of basal ganglia | Read |
| G660. | Middle cerebral artery syndrm | Read |
| G661. | Anterior cerebral artery syn | Read |
| G662. | Posterior cerebral artery syn | Read |
| G663. | Brain stem stroke syndrome | Read |
| G664. | Cerebellar stroke syndrome | Read |
| G665. | Pure motor lacunar syndrome | Read |
| G666. | Pure sensory lacunar syndrome | Read |
| G6760 | Cere infct/cere vn thrm,nonpyo | Read |
| G6W.. | Cer inf,un oc/st precer art | Read |
| G6X.. | Cereb in/uns oc,stn/cereb a | Read |
| Gyu63 | [X]Cereb in/uns oc,stn/cereb a | Read |
| Gyu64 | [X]Other cerebral infarction | Read |
| Gyu65 | [X]Oc+steno/o precerebral artr | Read |
| Gyu66 | [X]Oc+sten/o cerebral arteries | Read |
| Gyu6G | [X]Cer inf,un oc/st precer art | Read |
| Fyu55 | [X]Oth cerebral TIA's+rel synd | Read |
| G65.. | Transient cerebral ischaemia | Read |
| G650. | Basilar artery syndrome | Read |
| G651. | Vertebral artery syndrome | Read |
| G6510 | Vertebro-basilar artery syndrm | Read |
| G652. | Subclavian steal syndrome | Read |
| G653. | Carotid artery syn hemispheric | Read |
| G654. | Multi&bilat precerebrl art syn | Read |
| G656. | Vertebrobasilar insufficiency | Read |
| G657. | Carotid terr trans ischaem att | Read |
| G65y. | Other transient cerebral isch. | Read |
| G65z. | Transient cerebral ischaem.NOS | Read |
| G65zz | Transient cerebral ischaem.NOS | Read |
| ZV12D | [V]Pers hist trans isch attack | Read |

Table 3l: Cerebrovascular disease – haemorrhagic

| **Diagnostic code** | **Description** | **Code type** |
| --- | --- | --- |
| I600 | Subarachnoid haemorrhage from carotid siphon and bifurcation | ICD10 |
| I601 | Subarachnoid haemorrhage from middle cerebral artery | ICD10 |
| I602 | Subarachnoid haemorrhage from anterior communicating artery | ICD10 |
| I603 | Subarachnoid haemorrhage from posterior communicating artery | ICD10 |
| I604 | Subarachnoid haemorrhage from basilar artery | ICD10 |
| I605 | Subarachnoid haemorrhage from vertebral artery | ICD10 |
| I607 | Subarachnoid haemorrhage from intracranial artery, unspecified | ICD10 |
| I608 | Other subarachnoid haemorrhage | ICD10 |
| I609 | Subarachnoid haemorrhage, unspecified | ICD10 |
| I610 | Intracerebral haemorrhage in hemisphere, subcortical | ICD10 |
| I611 | Intracerebral haemorrhage in hemisphere, cortical | ICD10 |
| I612 | Intracerebral haemorrhage in hemisphere, unspecified | ICD10 |
| I613 | Intracerebral haemorrhage in brain stem | ICD10 |
| I614 | Intracerebral haemorrhage in cerebellum | ICD10 |
| I615 | Intracerebral haemorrhage, intraventricular | ICD10 |
| I616 | Intracerebral haemorrhage, multiple localized | ICD10 |
| I618 | Other intracerebral haemorrhage | ICD10 |
| I619 | Intracerebral haemorrhage, unspecified | ICD10 |
| I620 | Subdural haemorrhage (acute)(nontraumatic) | ICD10 |
| I621 | Nontraumatic extradural haemorrhage | ICD10 |
| I629 | Intracranial haemorrhage (nontraumatic), unspecified | ICD10 |
| I690 | Sequelae of subarachnoid haemorrhage | ICD10 |
| I691 | Sequelae of intracerebral haemorrhage | ICD10 |
| I692 | Sequelae of other nontraumatic intracranial haemorrhage | ICD10 |
| G61.. | Intracerebral haemorrhage | Read |
| G610. | Cortical haemorrhage | Read |
| G611. | Internal capsule haemorrhage | Read |
| G612. | Basal nucleus haemorrhage | Read |
| G613. | Cerebellar haemorrhage | Read |
| G614. | Pontine haemorrhage | Read |
| G615. | Bulbar haemorrhage | Read |
| G616. | External capsule haemorrhage | Read |
| G618. | Intracerebrl haem,multip local | Read |
| G619. | Lobar cerebral haemorrhage | Read |
| G61X. | Intracer haem hemisph, unsp | Read |
| G61X0 | Left side intracereb haem unsp | Read |
| G61X1 | Right side intracer haem unsp | Read |
| G61z. | Intracerebral haemorrhage NOS | Read |
| Gyu62 | [X]Oth intracerebrl h'morrhage | Read |
| Gyu6F | [X]Intracer haem hemisph, unsp | Read |

Table 3m: Cerebrovascular disease – unspecified

| Diagnostic code | Description | Code type |
| --- | --- | --- |
| G667. | Left sided CVA | Read |
| G668. | Right sided CVA | Read |

Table 3n: Hypertension

| **Diagnostic code** | **Description** | **Code type** |
| --- | --- | --- |
| I119 | Hypertensive heart disease without (congestive) heart failure | ICD10 |
| I120 | Hypertensive renal disease with renal failure | ICD10 |
| I129 | Hypertensive renal disease without renal failure | ICD10 |
| I131 | Hypertensive heart and renal disease with renal failure | ICD10 |
| I139 | Hypertensive heart and renal disease, unspecified | ICD10 |
| I150 | Renovascular hypertension | ICD10 |
| I152 | Hypertension secondary to endocrine disorders | ICD10 |
| I158 | Other secondary hypertension | ICD10 |
| I159 | Secondary hypertension, unspecified | ICD10 |
| 662F. | Hypertension treatm. started | Read |
| 662G. | Hypertensive treatm.changed | Read |
| G672. | Hypertensive crisis | Read |
| Gyu2. | Other secondary hypertension | Read |
| Gyu21 | Hypertension secondary to other renal disorders | Read |
| L12.. | Beningn essential hypertension in pregnancy/childbirth/puerperium | Read |
| L120. | Beningn essential hypertension in pregnancy/childbirth/puerperium | Read |
| L120- | Beningn essential hypertension in pregnancy/childbirth/puerperium | Read |
| L1200 | Beningn essential hypertension in pregnancy/childbirth/puerperium | Read |
| L1201 | Beningn essential hypertension in pregnancy/childbirth/puerperium | Read |
| L1203 | Beningn essential hypertension in pregnancy/childbirth/puerperium | Read |
| L1204 | Beningn essential hypertension in pregnancy/childbirth/puerperium | Read |
| L120z | Beningn essential hypertension in pregnancy/childbirth/puerperium | Read |
| L121. | Renal hypertension in pregnancy/childbirth/puerperium | Read |
| L1210 | Renal hypertension in pregnancy/childbirth/puerperium unspecified | Read |
| L1211 | Renal hypertension in pregnancy/childbirth/puerperium – delivered | Read |
| L1212 | Renal hypertension in pregnancy/childbirth/puerperium – delivered with complications | Read |
| L1213 | Renal hypertension in pregnancy/childbirth/puerperium – not delivered | Read |
| L1214 | Renal hypertension in pregnancy/childbirth/puerperium with postnatal complication | Read |
| L121z | Renal hypertension in pregnancy/childbirth/puerperium NOS | Read |
| L122. | Other pre-existing hypertension in preg/childbirth/puerperium | Read |
| L1220 | Other pre-existing hypertension in preg/childbirth/puerperium unspec | Read |
| L1221 | Other pre-existing hypertension in preg/childbirth/puerperium – deliv | Read |
| L1223 | Other pre-existing hypertension in preg/childbirth/puerperium – not deliv | Read |
| L1224 | Other pre-existing hypertension in preg/childbirth/puerperium + p/n comp | Read |
| L122z | Other pre-existing hypertension in preg/childbirth/puerperium NOS | Read |
| L123. | Transient hypertension of pregnancy | Read |
| L1230 | Transient hypertension of pregnancy unspecified | Read |
| L1231 | Transient hypertension of pregnancy - delivered | Read |
| L1232 | Transient hypertension of pregnancy – delivered with postnatal complication | Read |
| L1233 | Transient hypertension of pregnancy – not delivered | Read |
| L1234 | Transient hypertension of pregnancy with post-natal complication | Read |
| L1235 | Gestational hypertension | Read |
| L1236 | Transient hypertension of pregnancy | Read |
| L123z | Transient hypertension of pregnancy NOS | Read |
| L127. | Pre‑eclampsia or eclampsia with pre‑existing pre-existing hypertension | Read |
| L1270 | Pre‑eclampsia or eclampsia with pre-existing hypertension unspecified | Read |
| L1271 | Pre‑eclampsia or eclampsia with pre-existing hypertension – delivered | Read |
| L1272 | Pre‑eclampsia or eclampsia with pre-existing hypertension – delivered with postnatal comp | Read |
| L1273 | Pre‑eclampsia or eclampsia with pre-existing hypertension – not delivered | Read |
| L1274 | Pre‑eclampsia or eclampsia with pre-existing hypertension with postnatal complications | Read |
| L127z | Pre‑eclampsia or eclampsia with pre-existing hypertension NOS | Read |
| L128. | Pre-existing hypertension complicating pregnancy, childbirth and puerperium | Read |
| L1280 | Pre-existing hypertension complicating pregnancy, childbirth and puerperium | Read |
| L1281 | Pre-existing hypertensive heart and renal disease complicating pregnancy, childbirth and the puerperium | Read |
| L1282 | Pre-existing secondary hypertension complicating pregnancy, childbirth and puerperium | Read |
| L12B. | Proteinuric hypertension of pregnancy | Read |
| L12z. | Unspecified hypertension in pregnancy/childbirth/puerperium | Read |
| L12z0 | Unspecified hypertension in pregnancy/childbirth/puerperium unspecified | Read |
| L12z1 | Pregnancy-induced hypertension | Read |
| L12z2 | Unspecified hypertension in pregnancy/childbirth/puerperium – del + p/n comp | Read |
| L12z3 | Unspecified hypertension in pregnancy/childbirth/puerperium – not deliv | Read |
| L12z4 | Unspecified hypertension in pregnancy/childbirth/puerperium with p/n comp | Read |
| L12zz | Unspecified hypertension in pregnancy/childbirth/puerperium NOS | Read |
| Lyu1. | [X]Oedema, proteinuria+hypertension in pregnancy, childbirth puerperium | Read |
| Q000. | Fetus or neonate affected by maternal hypertensive disease | Read |
| SLC6. | Other hypertensive agent poisoning | Read |
| SLC6z | Hypertensive agent poisoning NOS | Read |
| SyuFT | Poisoning by antihypertensive agent | Read |
| TJC7. | Adverse reaction to other antihypertensives | Read |
| TJC7z | Antihypertensive adverse reaction | Read |
| U60C5 | [X]Oth antihyperten drug caus advers eff in therap use, NEC | Read |
| 6624 | Yearly observation of borderline hypertension | Read |
| 6627 | Good hypertension control | Read |
| 6628 | Poor hypertension control | Read |
| 6629 | Hypertension: follow-up default | Read |
| 61462 | Hypertension induced by oral contraceptive pill | Read |
| 14A2. | H/O: hypertension | Read |
| 1JD.. | Suspected hypertension | Read |
| 661M6 | Hypertension self-management plan agreed | Read |
| 661N6 | Hypertension self-management plan review | Read |
| 662-2 | Hypertension monitoring | Read |
| 662b. | Moderate hypertension control | Read |
| 662c. | Hypertension 6 month review | Read |
| 662d. | Hypertension annual review | Read |
| 662F. | Hypertension treatm. started | Read |
| 662G. | Hypertensive treatm. started | Read |
| 662H. | Treatment for hypertension stopped | Read |
| 662O. | On treatment for hypertension | Read |
| 662P. | Hypertension monitoring | Read |
| 662P0 | Hypertension 9 month review | Read |
| 662P1 | Telehealth hypertension monitoring | Read |
| 662q. | Trial reduction of antihypertensive therapy | Read |
| 662r. | Trial withdrawal of antihypertensive therapy | Read |
| 66b2. | Hypertension monitoring not required | Read |
| 67H8. | Lifestyle advice regarding hypertension | Read |
| 7Q01. | High cost hypertension drugs | Read |
| 7Q01y | Other specified high cost hypertension drugs | Read |
| 7Q01z | High cost hypertension drugs NOS | Read |
| 8B26. | Antihypertensive therapy | Read |
| 8BL0. | Patient on maximal tolerated antihypertensive therapy | Read |
| 8CR4. | Hypertension clinical management plan | Read |
| 8HT5. | Referral for hypertension clinic | Read |
| 8I3N. | Hypertension treatment refused | Read |
| 8IA5. | Trial withdrawal of antihypertensive therapy declined | Read |
| 8IA6. | Trial reduction of antihypertensive therapy declined | Read |
| 8OAH. | Provision of written information about diabetes and hypertension | Read |
| 9h3.. | Exception reporting - hypertension quality indicators | Read |
| 9h31. | Excepted from hypertension quality indicators - patient unsuitable | Read |
| 9h32. | Excepted from hypertension quality indicators - informed dissent | Read |
| 9N03. | Seen in hypertension clinic | Read |
| 9N1y2 | Seen in hypertension clinic | Read |
| 9N4L. | DNA - Did not attend hypertension clinic | Read |
| 9OI.. | Hypertension monitoring administration | Read |
| 9OI1. | Attends hypertension monitoring | Read |
| 9OI2. | Hypertension monitoring refused | Read |
| 9OI3. | Hypertension monitoring offer default | Read |
| 9OI4. | Hypertension monitoring first letter | Read |
| 9OI5. | Hypertension monitoring second letter | Read |
| 9OI6. | Hypertension monitoring third letter | Read |
| 9OI7. | Hypertension monitoring verbal invite | Read |
| 9OI8. | Hypertension monitoring telephone invite | Read |
| 9OI9. | Hypertens.monitor deleted | Read |
| 9OIA. | Hypertension monitoring check done | Read |
| 9OIB. | Borderline hypertension monitoring first letter | Read |
| 9OIC. | Borderline hypertension monitoring second letter | Read |
| 9OID. | Borderline hypertension monitoring third letter | Read |
| 9OIZ. | Hypertension monitoring. Admin. NOS | Read |
| G2... | Hypertensive disease | Read |
| G21.. | Bening hypertensive heart disease | Read |
| G20.. | Essential hypertension | Read |
| G201. | Benign essential hypertension | Read |
| G202. | Systolic hypertension | Read |
| G200. | Malignant essential hypertension | Read |
| G203. | Diastolic hypertensionypertension | Read |
| G20z. | Essential hypertension NOS | Read |
| G21.. | Hypertensive heart disease | Read |
| G210. | Malignant hypertensive heart disease | Read |
| G2100 | Malignant hypertensive heart disease without CCF | Read |
| G2101 | Malignant hypertensive heart disease with CCF | Read |
| G210z | Malignant hypertensive heart disease NOS | Read |
| G211. | Beningnhypertensive heart disease | Read |
| G2110 | Beningnhypertensive heart disease with CCF | Read |
| G2111 | Beningnhypertensive heart disease without CCF | Read |
| G211z | Beningnhypertensive heart disease NOS | Read |
| G21z. | Hypertensive heart disease NOS | Read |
| G21z0 | Hypertensive heart disease NOS without CCF | Read |
| G21z1 | Hypertensive heart disease NOS with CCF | Read |
| G21zz | Hypertensive heart disease NOS | Read |
| G22.. | Hypertensive renal disease | Read |
| G220. | Malignant hypertensive renal disease | Read |
| G221. | Benign hypertensive renal disease | Read |
| G222. | Hypertensive renal disease with renal failure | Read |
| G22z. | Hypertensive renal disease NOS | Read |
| G23.. | Hypertensive heart and renal disease | Read |
| G230. | Malignant hypertensive heart and renal disease | Read |
| G231. | Benign hypertensive heart and renal disease | Read |
| G232. | Hypertensive heart and renal disease with heart failure | Read |
| G233. | Hypertensive heart and renal disease with renal failure | Read |
| G234. | Hypertensive heart and renal disease with heart and renal failure | Read |
| G23z. | Hypertensive heart and renal disease NOS | Read |
| G24.. | Secondary hypertension | Read |
| G240. | Secondary malignanthypertension | Read |
| G2400 | Secondary malignant reno vascular hypertension | Read |
| G240z | Secondary malignant hypertension NOS | Read |
| G241. | Secondary benign hypertension NOS | Read |
| G2410 | Secondary benign renovascular hypertension | Read |
| G241z | Secondary benign renovascular hypertension NOS | Read |
| G244. | Hypertension secondary to endocrine disorders | Read |
| G24z. | Secondary hypertension NOS | Read |
| G24z0 | Secondary renovascular hypertension NOS | Read |
| G24z1 | Hypertension secondary to a drug | Read |
| G24zz | Secondary hypertension NOS | Read |
| G25.. | Stage 1 hypertension (NICE) | Read |
| G250. | Stage 1 hypertension (NICE, 2011) without evidence of end organ damage | Read |
| G251. | Stage 1 hypertension (NICE, 2011) with evidence of end organ damage | Read |
| G26.. | Severe hypertension (NICE 2011) | Read |
| G26-1. | Severe hypertension | Read |
| G27.. | Hypertension resistant to drug therapy | Read |
| G28.. | Stage 2 hypertension (NICE) | Read |
| G2y.. | Other specified hypertensive disease | Read |
| G2z.. | Hypertensive disease NOS | Read |

Table 3o: Fatty liver disease (now known as MASD liver disease)

| **Diagnostic code** | **Description** | **Code type** |
| --- | --- | --- |
| K758 | Non-alcoholic Steatohepatitis | ICD10 |
| K760 | Fatty liver (NOS) | ICD10 |

Table 4: Frequency counts of first codings for co-morbidities throughout follow-up. Note drop-outs and death throughout follow-up are accounted for in the time-to-event analysis and hypothesis testing with the log-rank test provided in the Results section of the main manuscript.

| Co-morbidity | Non-diabetic  (*n =* 64, 709) | GDM  (*n =* 400) |
| --- | --- | --- |
| Hypertension | 4225 | 48 |
| Eye complications | 1762 | 75 |
| Fatty Liver | 1056 | 27 |
| IHD | 939 | 12 |
| Heart failure | 101 | 5 |
| CVD – ischaemic | 93 | 0 |
| CVD – unspecified | 49 | 0 |
| CVD – haemorrhagic | 35 | 1 |
| Neuropathy | 44 | 2 |
| Cataract | 44 | 0 |
| Vascular disease | 56 | 0 |
| Diabetic foot disease | 5 | 0 |
| Amputation | 0 | 0 |
